# Down-Regulation of P450 Genes Enhances Susceptibility to Indoxacarb and Alters Physiology and Development of Fall Armyworm, *Spodoptera frugipreda* (Lepidoptera: Noctuidae)

**DOI:** 10.3389/fphys.2022.884447

**Published:** 2022-05-09

**Authors:** Muhammad Hafeez, Xiaowei Li, Farman Ullah, Zhijun Zhang, Jinming Zhang, Jun Huang, G. Mandela Fernández-Grandon, Muhammad Musa Khan, Junaid Ali Siddiqui, Limin Chen, Xiao Yun Ren, Shuxing Zhou, Yonggen Lou, Yaobin Lu

**Affiliations:** ^1^ State Key Laboratory for Managing Biotic and Chemical Threats to the Quality and Safety of Agro-products, Institute of Plant Protection and Microbiology, Zhejiang Academy of Agricultural Sciences, Hangzhou, China; ^2^ State Key Laboratory of Rice Biology and Ministry of Agriculture Key Lab of Molecular Biology of Crop Pathogens and Insects, Institute of Insect Sciences, Zhejiang University, Hangzhou, China; ^3^ Department of Plant Biosecurity, College of Plant Protection, China Agricultural University, Beijing, China; ^4^ Natural Resources Institute, University of Greenwich, Greenwich, United Kingdom; ^5^ Key Laboratory of Bio-Pesticide Innovation and Application, South China Agricultural University, Guangzhou, China; ^6^ Red Imported Fire Ant Research Centre, South China Agricultural University, Guangzhou, China; ^7^ Integrated Plant Protection Center, Lishui Academy of Agricultural and Forestry Sciences, Lishui, China

**Keywords:** Fall armyworm, Detoxification enzymes, P450 genes, indoxacarb resistance, RNAi

## Abstract

The fall armyworm (FAW), *Spodoptera frugiperda* (J.E. Smith), is a pest of many important crops globally. Effective control is challenging, with the pest exhibiting resistance to different synthetic pesticides across various groups. However, the mechanisms employed by resistant insects for overexpression of relevant detoxification genes remain unclear. The activity of detoxification enzymes was investigated in this study. Additionally, using RNA interference (RNAi), a functional analysis was completed of two P450s genes in an indoxacarb resistant population of fall armyworms. Elevated resistance levels (resistance ratio = 31.37-fold) in indoxacarb-selected populations of FAW were observed after 14 generations. The qRT-PCR showed higher expression of two cytochrome P450 genes, *CYP321A7* and *CYP6AE43*, in this selected population compared to the control population. RNAi was applied to knock down the P450 ds*CYP321A7* and ds*CYP6AE43* genes in the FAW larvae. Droplet feeding of the dsRNAs (*CYP321A7* and *CYP6AE43*) via an artificial diet significantly increased mortality rates in the indoxacarb treated population. A shorter larval developmental time of FAW was detected in all dsRNAs-fed larvae. Correspondingly, larval mass was reduced by dsRNAs in indoxacarb resistant populations of fall armyworm. Larval feeding assays demonstrate that dsRNAs targeting, specifically of *CYP321A7* and *CYP6AE43* enzymes, could be a beneficial technique in the management of indoxacarb resistant populations. Further study on the potential use of dsRNA and its application should be conducted in efforts to counter the development of resistance in FAW against various insecticides in the field.

## Introduction

The fall armyworm (FAW), *Spodoptera frugiperda* (J.E. Smith), is an invasive polyphagous insect pest, causing serious damage to major crops such as corn, rice, sorghum, and peanut (Goergen et al., 2016; Montezano et al., 2018). This pest, which originated in the Americas, has spread rapidly around the world, causing significant crop losses in each new location where it has been discovered (Goergen et al., 2016). FAW has spread in West and Central Africa in recent years, and it has recently been discovered in Indonesia and Southwest China (Southwest Yunnan province) (Goergen et al., 2016; Ginting et al., 2020; Li et al., 2020). FAW has the potential to cause 8.3 to 20.6 million tons of corn losses across Africa each year, according to the International Centre for Agricultural and Biological Sciences (Day et al., 2017).

Traditionally, various synthetic insecticides have been used to control insect pests in major crops (Desneux et al., 2007; Burtet et al., 2017; Hafeez et al., 2019b; Gul et al., 2021). Currently, the application of synthetic insecticides remains the primary approach to control FAW in China. However, the long-term use in large quantities of chemical insecticides could easily lead to resistance strains developing as well as create serious problems for human health, destruction of non-target organisms, hormesis and further environmental pollution (Desneux et al., 2006; Davis 1993; Aktar et al., 2009; Nawaz et al., 2018; Hafeez et al., 2019c; Ullah et al., 2019; Iftikhar et al., 2020). These issues are exacerbated once pests have developed resistance to large groups of insecticides, which directly reduces their efficacy (Mallet 1989; Zhang et al., 2020; Ullah et al., 2021). In the field, insecticides such as indoxacarb, chlorantraniliprole, emamectin benzoate, and *Bacillus thuringiensis* are mainly used to control lepidopteran pests (Cordova et al., 2006; Xiao et al., 2016; Wang et al., 2019; Hafez et al., 2020; Zhang et al., 2021). The genetic malleability and the exhaustive selection pressure of insecticide applications has led insect pests such as *Plutella xylostella*, *Mythimna separate, Choristoneura rosaceana* and *S. frugiperda* to develop effective resistance to chlorantraniliprole, emamectin benzoate, indoxacarb and *Bacillus thuringiensis* (Wang et al., 2013, 2019; Monnerat et al., 2015; Liu et al., 2016; Hafez et al., 2020). Indoxacarb is an oxadiazine insecticide discovered in 1992 by the E.I. DuPont Co. and commercialized to the open market in 2000 (McCann et al., 2001; Thompson and Dutton, 2003). Indoxacarb is highly active when ingested, but there have been few reports of contact activity when applied topically (Alves et al., 2008; Nehare et al., 2010). This is the premise for indoxacarb’s exceptional activity against several lepidopteran species, regardless of the fact that some field populations of major insect pests, including FAW, have evolved a high level of resistance to indoxacarb (Wing et al., 2000; Ahmad et al., 2008; Hafeez et al., 2020; Zhang et al., 2021). To develop effective FAW management practices, we must first understand the mechanisms underlying their resistance to these insecticides.

Insect resistance to synthetic insecticides is an ongoing challenge to sustainable pest management while also providing a suitable model system to study adaptive evolution. One of the molecular alterations frequently implicated in resistant insect populations is the increased production of metabolic enzymes that detoxify or sequester insecticides before they reach the target site (Feyereisen, 2012; Yang et al., 2020). High metabolic activity of detoxifying enzymes such as cytochrome P450 monooxygenase, esterases (Ests), and glutathione *S*-transferase (GSTs) has been identified as among the key mechanisms that underlie insecticide resistance (Li et al., 2007; Li and Liu, 2017). A key enzyme system in this regard is insect cytochrome P450s, which play a central role in the metabolism of a wide range of natural and synthetic xenobiotics, including insecticides, and have been classified into four major clades: CYP2, CYP3, CYP4, and the mitochondrial CYP (Scott, 1999a; Feyereisen, 2012). Among them, the clade *CYP3* is further subdivided into different CYP families and subfamilies. The subfamilies *CYP321* and *CYP6* of clade *CYP3* are known to play a significant role in the metabolism of xenobiotics and, accordingly, insecticide resistance (Feyereisen, 2006a; Li et al., 2007). Induction of P450 genes is commonly seen in insects and may be initiated by a variety of compounds found in their environment. For example, research has shown that increased metabolic enzyme activity caused by increased mRNA expression levels of detoxification genes facilitates insecticide resistance in insects (Vontas et al., 2000; Arain et al., 2018; Hafeez et al., 2019c). In Lepidoptera, numerous key P450 genes such as *CYP6AB14* and *CYP9A98* in *Spodoptera exigua*, *CYP9A14*, *CYP337B1*, *CYP9A12*, *CYP6AE11* and *CYP6B7* in *Helicoverpa armigera,* and *CYP321A8*, *CYP321A9*, and *CYP321B1* in *Spodoptera frugiperda* were frequently identified as being associated with various insecticide resistance mechanisms (Hafeez et al., 2019d; Wee et al., 2008; Zhao et al., 2014; Bai-Zhong et al., 2020; Tang et al., 2020). As a result, it is critical to investigate the induction effect of this insecticide on FAW to assess its potential for use in pest control.

RNAi, which uses gene silencing based on the conserved biological defense response at the cellular level triggered by double-stranded RNA (dsRNA), is a promising technology in agriculture. This method could pave the way for the next generation of insect-resistant GM crops (Mello and Conte, 2004; Zhang et al., 2017; Chen et al., 2019; Bennett et al., 2020). The roles of multiple P450 genes of various insects in insecticide resistance and phytochemical detoxification have been functionally confirmed by the induction experiments. These demonstrate the increased larval sensitivity to toxins after RNAi-mediated silencing of specific P450 genes (Sun et al., 2019; Lu et al., 2020; Wang et al., 2022). When dsRNA is ingested via an artificial diet or through droplet feeding, it can knock down genes through the RNAi pathway, resulting in a reduction in the target pest’s growth and mortality (Ehrlich and Raven 1964; Ahmad et al., 2008; Lim et al., 2016; Zhang et al., 2017; Hafeez et al., 2019e; Ullah et al., 2020a).

The primary goal of this study was to identify the expression and function of P450 genes in an FAW population that has developed significant levels of resistance to indoxacarb in the laboratory. Two up regulated P450 genes (*CYP321A7* and *CYP6AE43*) related to resistance were selected to study the expression and function in an indoxacarb resistant population of FAW. These findings provide a better understanding of the molecular functions of P450 genes and insecticide resistance mechanisms, findings that may facilitate future pest management strategies for FAW.

## Materials and Methods

### Insect Collection and Rearing

In August 2019, 200 fall armyworm larvae of various instars were collected from two different cornfields in Ping Hu, Zhejiang province, to establish a laboratory population. Larvae were raised on a semi-solid artificial diet based on pinto bean powder, as previously described (Zhao et al., 2020). They were kept at 25 ± 2°C on a 14:10 h light: dark photoperiod in a climate control chamber. Following pupation, newly hatched adults were segregated into mating pairs and fed a 10% sugar solution as an additional food source. Two generations were reared before conducting selection bioassays. The population was divided into two subpopulations: those exposed to no insecticide treatment denoted as Indox-UNSEL and those exposed to indoxacarb denoted as Indox-SEL. After 14 generations of selection with indoxacarb, the Indox-SEL population were identified as a resistant strain.

### Chemicals

Indoxacarb 15% (commercial formulation) was purchased from Mesa Tech International Inc (China). 7-ethoxycoumarin and 7-hydroxycoumarin were bought from Sigma-Aldrich (St. Louis, MO, United States). The synergist, piperonyl butoxide (PBO) was obtained from Shanghai Aladdin Bio-chem Technology Co., Ltd (China). Bovine serum albumin was purchased from Beyotime Biotechnology (Jiangsu, China).

### Toxicity Bioassays

The toxicity bioassay at generation one (G1) on two-day-old second-instar larvae was conducted using the previously described diet incorporation method (Hafeez et al., 2019a). In brief, six concentrations of insecticide were diluted from the stock (via serial dilutions) with distilled water including a control (without insecticide). In each concentration, there were four replicates and the semisynthetic diet was thoroughly mixed using a well-established method (Gupta et al., 2005). The required concentrations were mixed gently before the agar solidified (40–45°C), then placed into new sterile transparent plastic cups to prevent cross-contamination (3 cm diameter, 3.5 cm height). The artificial diet without insecticide was established as a control treatment. Ten two-day-old second-instar larvae were placed in each transparent plastic cup containing an insecticide-supplemented diet. Four replicates were completed for each, providing a total of 40 larvae per concentration. Similarly, the control treatment was performed with larvae exposed to the artificial diet without insecticide. Control mortality was less than 10%. The bioassays were kept in a climate control chamber at 25 ± 2°C (14:10 h) light: dark photoperiod with 60 ± 5% RH. Mortality was evaluated 72 h after exposure to indoxacarb. Larvae that did not move after being touched with a fine paintbrush were deemed dead.

### Resistance Selection of FAW to Indoxacarb

The selection with indoxacarb from generations 1–14 of *S. frugiperda* was done by a diet incorporation method as previously described to create the Indox-SEL population. From generation 1 to generation 14, two-day-old second-instar larvae were exposed to various concentrations (4–110 μg g^−1^) throughout their rearing. The field-collected population of FAW developed high levels of resistance to indoxacarb after 14 generations of continuous selection. The LC_50_ value was calculated 72 h after treatment to indoxacarb and the surviving larvae of every selection were raised on an artificial diet to obtain the next generation. Two-day-old second-instar larvae selected per generation ranged from 200 to 300 individuals. The laboratory reared population without exposure to any insecticide, Indox-UNSEL, was used as a reference strain for resistance monitoring.

### Synergism Bioassay

To evaluate the metabolic resistance to indoxacarb, the larvae were exposed to known pesticide synergists, piperonyl butoxide (PBO), triphenyl phosphate (TPP) and diethyl maleate (DEM) then subjected to the test insecticide. Synergistic mechanisms associated with PBO, TPP and DEM were evaluated using a previously described method (Zhao et al., 2020). Solutions of PBO, TPP and DEM at the concentration of 50 mg/L, 50 mg/L and 100 mg/L were prepared in 1% (v/v) acetone. For both Indox-SEL and Indox-UNSEL groups, acetone solutions (1 μL) of PBO TPP and DEM were applied to the pronotum of individual third instar larvae using a hand applicator. Larvae were left for 2 h before indoxacarb treatment. There was no mortality in FAW after exposure to any of these synergists. The FAW larvae were then moved into the transparent plastic cup containing an indoxacarb-treated diet of different concentrations for 72 h. There were two control groups: one exposed and then fed an artificial diet containing 1% (v/v) acetone, and the other exposed and then fed an artificial diet only. All population were maintained in a controlled environment matching their rearing conditions.

### Measurement of Enzyme Activity

#### Sample Preparation

The detoxification enzyme activity of P450 and esterase in the midgut homogenates of the Indox-SEL and Indox-UNSEL populations was measured. For the Indox-SEL population, late third-instar larvae of a similar size were selected and transferred into transparent plastic cups containing diets supplemented with an LC_20_ dose of indoxacarb. The Indox-UNSEL population was fed on control diet without insecticide for 48, 72, and 96 h. The midguts from treated (Indox-SEL population) and untreated (Indox-UNSEL population) groups were dissected and gently shaken to release their contents before being washed in a cold aqueous solution. The crude homogenates of FAW midguts from treated and untreated groups were prepared according to Liu et al*.* (2006). It was done in biological triplicate for every treatment that was given.

### P450 Enzyme Activity Measurement

Evaluation P450 enzyme activity followed the methodology from Chen et al*.* (2018), with slight modification. A total of 100 µL of 2 mM p-nitroanisole solution was put into each well of a clear 96-well plate containing 90 µL of crude enzyme, and the microplate was incubated for 3 min at 27°C before adding 10 µL of 9.6 mM NADPH to initiate the reaction. The absorbance was measured using a microplate reader. The activity was recorded as nmol p-nitroanisole/min/mg protein.

Protein concentration was determined using the Bradford method (Bradford, 1976).

### Measurement of the Activity of Esterase

The activity of the esterase (EST) enzyme was determined using established methodologies (van Asperen, 1962; Wu et al., 2011). A total of 200 µL of substrate solution (0.1 ml 100 mM a-NA, 10 mg 10 mg Fast Blue RR salt, and 5 ml 0.2 M pH 6.0 phosphate buffer) and 10 µL enzyme solution were gently mixed and added to each well. Enzyme activity was measured using an xMark Microplate Spectrophotometer (BIO-RAD) and recorded every 15 s. The activity of the enzyme was denoted as nmol a-naphthol/min/mg protein.

### Extraction of RNA and Preparation of cDNA

Trizol reagent (Takara, Japan) was used to extract total RNAs from different tissues of larvae and adults (male and female) of Indox-UNSEL and Indox-SEL populations after 72 h following the manufacturer’s protocol. First-strand complementary DNA (cDNA) was synthesized by using TransScript^®^ One-Step gDNA Removal and cDNA Synthesis SuperMix in 20 µL reactions containing 1 µg of total RNA (500 ng), 1 µL Anchord Oligo (dT)18 Prime (0.5 µg/uL), 10 µL 2xTS Reaction mixture, TransScript^®^ RT/RI EnzymeMix and gDNA Remover at 42°C for 30 min and preserved until use.

### Expression Analysis of P450 Genes in Indox-UNSEL and Indox-SEL Populations

qRT-PCR was performed using a CFX Connect TM Real-Time System (Bio-Rad, United States) to check the expression patterns of selected P450 genes. The RT-qPCR reaction mixtures contained 10 µL SsoFast EvoGreen^®^ qPCR SuperMix, 0.5 μL forward and reverse primers (10 μM each), 1 μL of cDNA template, and nuclease free water to a total volume of 20 μL. The thermo cycling protocol used was 94°C for 3 min, followed by 40 cycles of 94°C for 15 s, 57–60°C for 30 s and 70°C for 30 s. The gene sequences were downloaded from NCBI and primers were designed using Primer Premier 5 software (Premier Biosoft, United States) (Supplementary Table S1). The mean expression of the two reference genes, GAPDH (KC262638.1) and ribosomal protein S30 (AF400225.1) were used for data normalization according to (Bustin et al., 2009) (Supplementary Table S1). The 2^−ΔΔCt^ method described by Livak & Schmittgen (2001) was used to estimate mRNA expression levels. Three biological and three technical replicates were used in the qRT-PCR analysis.

### Bioinformatic and Phylogenetic Analysis of Selected P450 Genes

The protein sequences of all selected P450 genes from *CYP3*, *CYP2*, *CYP4* and Mito-clade were aligned using DNAMAN software. MEGA 7 software was used to construct a neighbour-joining tree with the Minimum-Evolution method (1000 bootstrap replications). The protein sequence was queried against the pdb database to find a template in NCBI blast. Then against both queries, 5cd1 was found to be highly homozygous with more than 30 percent sequence similarity. The modelling was performed using modeller 9.1.

### dsRNA Preparation and Feeding Bioassays

For dsRNA synthesis, The ORF and the conserved domains of *CYP321A7* and *CYP6AE43* sequences were found using NCBI. Primer Premier 5 software (Premier Biosoft, United States) was used to design primers with respective fragment sizes of 506 bp and 679 bp and were amplified by PCR. In addition, pGEM T-easy plasmid carrying the *dsRED* gene with fragment sizes of 421 bp was used as the template for PCR of the products to synthesize dsRNA (Jan et al., 2017). The primers for the targets (*CYP321A7* and *CYP6AE43*) and *dsRED* as a control gene amplification were designed using the T7 polymerase promoter sequence at the 5 ends of each strand (Table 1). To prepare dsRNA, we used the purified PCR-generated templates from *T7-CYP331A7*, *T7-CYP6AE43*, and T7-RED using the T7 RiboMAX Express RNAi System (Promega, Madison, WI, United States). MEGA clearTM Kit (Ambion) was used for the purification of dsRNA. The dsRNA was resuspended in diethyl pyrocarbonate (DEPC)-treated water to a final concentration of (500 ng/μL) and stored at 80°C for further use. In line with previous work, droplet-feeding was used for dsRNA feeding bioassays (Wang et al., 2018a; Hafeez et al., 2019c). The DEPC-treated water was used to prepare the dsRNA solution (500 ng/μL). The larvae were given 0.5 μL of dsRNA solution in droplets. A preliminary trial was performed to establish the effect of dsRNAs on mortality and development of FAW larvae. The artificial diet (1 g) was poured into each well of the sterilized 24-orifice tissue culture plate before solidification. A single drop of dsRNA solution 0.5 µL (500 ng/μL) was placed at the center of each well of diet using a 2 µL pipette (www.eppendorf.com) for 24 h followed by the exposure to an artificial diet supplemented with 6.84 μg g^−1^ of indoxacarb after 48, 72 and 96 h. The same method was applied for the dsRED as a control group. The artificial diet + DEPC without exposure to indoxacarb was also used as a control. Each treatment consisted of sixty starved larvae, three replicates of 20 larvae each. The mortality data were recorded at 48, 72 and 96 h. To evaluate the efficacy of RNAi-mediated knockdown of two selected P450 genes, the midguts were dissected from larvae that fed on dsRNAs (*dsCYP332A7* and *dsCYP6AE4*3, *dsRED*, and DEPC-water) for 24 h followed by the exposure to an artificial diet supplemented with 6.84 μg g^−1^ of indoxacarb after 48, 72 and 96 h for RNA extraction and RT-qPCR analysis.

**TABLE 1:** Primers used in this study for RNAi.

Primers Used for RNAi	Sense Primers	Anti-sense Primers	—
dsCYP6AE43	5′-gga​tcc​taa​tac​gac​tca​cta​tag​g GAT​ATG​CTG​AAC​GCC​GAC​CT-3′	5′-gga​tcc​taa​tac​gac​tca​cta​tag​g CTA​TCA​CCG​GGT​ACA​TCC​GC-3	679
dsCYP321A7	5′-gga​tcc​taa​tac​gac​tca​cta​tag​g GCT​ACT​GGA​AAA​AGC​GTG​GC-3′	5′-gga​tcc​taa​tac​gac​tca​cta​tag​gTG​AGT​TCG​TTC​CAA​TGC​CGA-3′	506
dsRED	5′-gga​tcc​taa​tac​gac​tca​cta​tag​g GCA​AGC​TAT​GCA​TCC​AAC​GCG​TTG​GG-3′	5′-gga​tcc​taa​tac​gac​tca​cta​tag​g CAA​GCT​ATG​CAT​CCA​ACG​CGT​TGG​GAG-3′	421
S30	CAC​CCT​CGG​TGT​TAG​ACG​TT	CCA​CCG​GGA​AAG​TGA​TAC​TGT	119
GAPDH	CGG​TGT​CTT​CAC​AAC​CAC​AG	TTG​ACA​CCA​ACG​ACG​AAC​AT	111

### The Single and Combined Effects of ds*CYP332A1* and ds*CYP6AE43* on Mortality and Development

To examine the combined effect of the two target genes, 250 ng of each dsRNA was used (ds*CYP332A7* + ds*CYP6AE43*). The droplet-feeding method described above was used to administer dsRNA in all treatments.

The larval mortality, developmental time, and weight were observed after feeding larvae on dsRNAs for 24 h. A total of 60 FAW larvae from each treatment (three replicates each of 20 larvae) were individually transferred into a 24-orifice tissue culture plate containing artificial diet supplemented with indoxacarb LC_50_ as described above. All tests were done in triplicate. All treatment groups recorded mortality at 48, 72, and 96 h, and larval weight at 72 h after feeding on dsRNA of each target gene. The surviving larvae were used to estimate the larval developmental period, larval weight, midgut physiology and pupal duration. Three triplicates were used for each treatment in all experiments. Midguts of larvae were dissected after 72 h of treatment with dsRNAs and control. The physiology of the midgut from each treated and control larva was observed under the microscope (Olympus, SZX2-ILLK, Tokyo, Japan) using a digital camera fed into a computer.

### Statistical Analysis

The lethal concentrations from the mortality bioassay data were estimated using Polo-PC software (Robertson and Preisler, 1992; Hafeez et al., 2019a). Data related to all experiments and the relative mRNA expression levels of P450 genes, were analyzed using SPSS 20.0 Software Package (SPSS Inc., Chicago, IL, United States).

## Results

### Toxicity of Indoxacarb to Indox-UNSEL and Indox-SEL Populations of FAW

The indoxacarb resistance in FAW was obtained by continuous selection of the population for 14 generations under laboratory conditions (Table 2). The unselected population (Indox-UNSEL) was found to have a lower LC_50_ (0.67 μg g^−1^) to indoxacarb than the selected population (Indox-SEL) which, after 14 generations of exposure and selection, had an LC_50_ (21.02 μg g^−1^) (Table 2). This high level of resistance is 31.37-fold compared to the unselected control group.

**TABLE 2:** Resistance to indoxacarb in field-collected populations of *S. frugiperda* after 14 generations of selection.

Insecticide	*N*	(µg-G-1) (95% CI)		Slope ±S.E.	*X* ^*2*^	*df*	Sr
		LC_50_	LC_20_				
Indox-UNSEL	630	0.67 (0.57 ± 0.79)	0.343 (0.24 ± 0.39)	2.03 ± 0.19	0.43	4	1
Indox-SEL (G-14)	630	21.02 (17.96 ± 24.38)	6.84 (5.04 ± 8.67)	1.88 ± 0.16	1.53	4	31.37

The 95% CI of RR were calculated according to Robertson and Preisler (1992) and considered significant if these did not include the value of 1. *N*: Number of individuals exposed was 630 insects and constant across all bioassays; degrees of freedom (df) was 4. CI = Confidence interval. RR = Resistance ratio a RR = LC_50_ value of insecticides of Indox-SEL-G14 divided by LC_50_ value of insecticides of Indox-UNSEL.

### Synergistic Assessment

An assay was conducted on the effect of indoxacarb synergists PBO, TPP and DEM on the Indox-UNSEL and Indox-SEL population (Table 3). The highest synergistic ratio for the Indox-SEL population was found for PBO (2.39), followed by TPP (at 1.31) and DEM (at 1.04). Results suggest that the cytochrome P450 may be involved in the detoxification mechanism of indoxacarb causing resistance in FAW (Table 3).

**TABLE 3:** Synergism by PBO, TPP and DEM in indoxacarb-treated larvae of *S. frugiperda*.

Insecticide	*N*	LC50 (µg-G-1) (95% CI)	Slope ±S.E.	SR^a^
Indoxacarb + UNSEL	630	0.67 (0.57 ± 0.79)	2.03 ± 0.19	—
Indoxacarb + PBO	315	0.549 (0.47 ± 0.67)	1.90 ± 0.16	—
Indoxacarb + TPP	315	0.58 (0.49 ± 0.68)	1.94 ± 0.17	—
Indoxacarb + DEM	315	0.785 (0.65 ± 0.92)	2.02 ± 0.20	—
Indoxacarb + SEL	630	21.02 (17.96 ± 24.38)	1.88 ± 0.16	—
Indoxacarb + PBO	315	8.81 (7.53 ± 10.16)	1.85 ± 0.15	2.39
Indoxacarb + TPP	315	16.11 (13.57 ± 18.90)	1.75 ± 0.16	1.31
Indoxacarb + DEM	315	20.13 (16.20 ± 23.70)	1.72 ± 0.16	1.04

a SR^a^ (synergism ratio) = LC_50_ of a population treated with indoxacarb alone divided by LC_50_ of the same population treated with indoxacarb plus a synergist.

### Detoxification Enzymes Activity of P450 and Esterase

**FIGURE 1:**
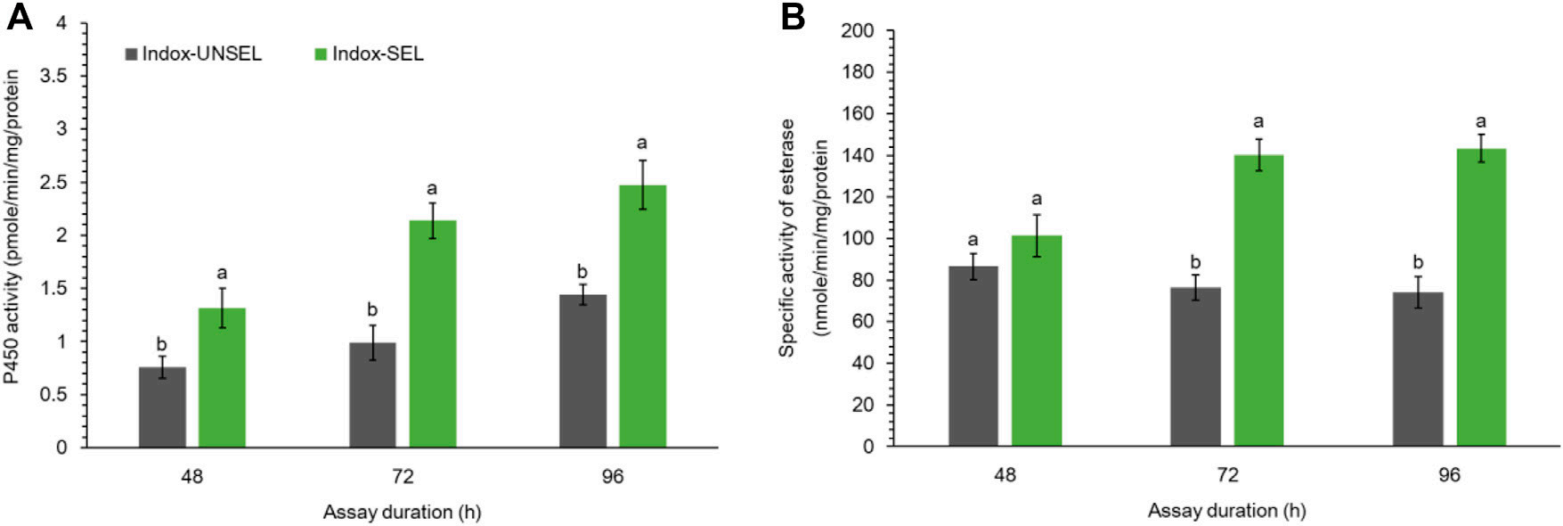
Activity of P450 enzyme **(A)** and specific activity of esterase **(B)** in midguts of the fourth-instar larvae of Indox-UNSEL and Indox-SEL population of FAW after 48, 72 and 96 h exposure to LC_20_ (6.84 μg g^−1^) concentration of indoxacarb. The data were expressed as the means ± SE. The bars with lowercase letters indicate significant differences within the same treatment time at the *p* < 0.05 level, based on Student’s *t* test.

Detoxification enzyme activity of P450 using 7-ethoxycoumarin (7-EC) as the substrate was assayed from the midgut of Indox-UNSEL and Indox-SEL populations (Figure 1A). The enzyme activity of cytochrome P450 with 7-EC substrate was significantly increased with time after an exposure to an LC_20_ (6.84 μg g^−1^) dosage of indoxacarb, whereas the highest P450 activity was noted after 96 h, in the midgut of the Indox-SEL larvae (Figure 1A). Similarly, higher activity of esterase was observed at 96 h for the Indox-SEL population after exposure to indoxacarb compared to the Indox-UNSEL population (Figure 1B).

### Expression pattern of P450s genes in the midgut of *S. frugiperda* and phylogenetic analysis

**FIGURE 2:**
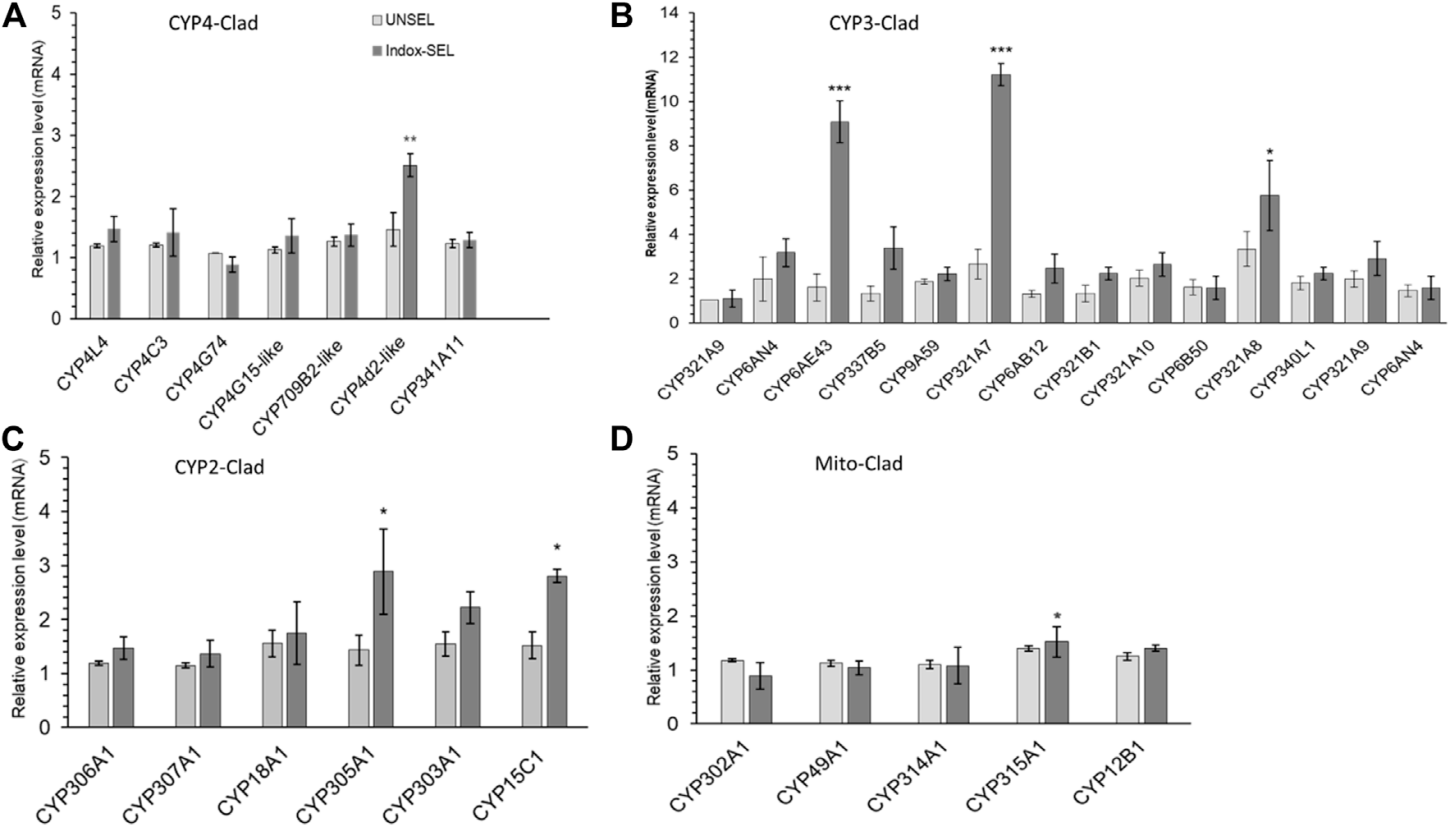
Midgut expression profiles of CYP4-clade **(A)**, CYP3-clade **(B)**, CYP2-clade **(C)** and Mito-clade **(D)** from *S. frugiperda* larvae. Bars represent relative expression (mean ± SE). All biological groups contained three replicates for each treatment, and there were three technical replicates. The transcription levels of all P450s genes determined by quantitative real-time PCR, normalized to two reference genes. The data were expressed as the means ± SE. The bars with astriks indicate significant differences within the same treatment at the *p* < 0.05 level, based on Student’s *t* test.

**FIGURE 3:**
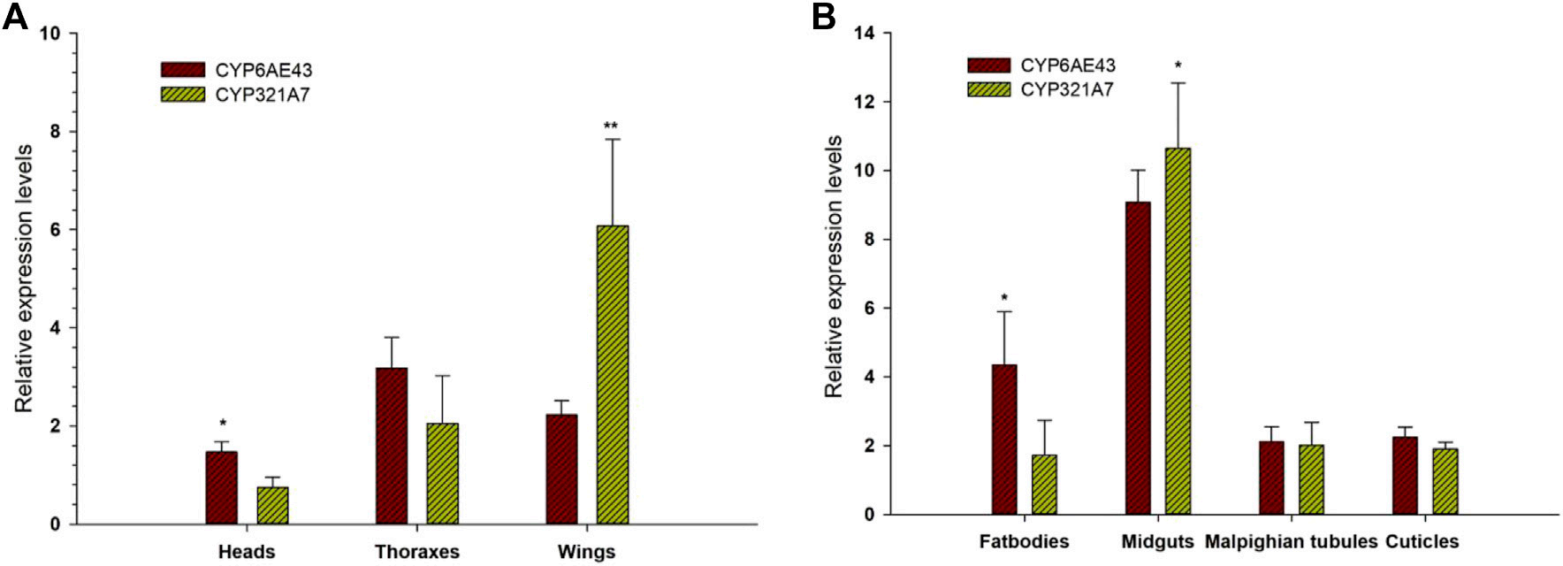
Tissue specific expression pattern of CYP6AE43 and CYP321A7 in midguts, bodies, malpighian tubules and cuticles of FAW larvae **(A)** and the heads, thoraxes and wings of adults **(B)**. Data shown are means ± SE derived from three biological replicates. Bars represent relative expression (mean ± SE). All biological groups contained three replicates for each treatment, and there were three technical replicates. The relative expression was calculated using the 2^−ΔΔCT^ method based on the value of the egg expression, which was ascribed an arbitrary value of 1. The astriks on the bar represent significant differences (*p* < 0.05) using one-way ANOVA, followed by Tukey’s HSD multiple comparison tests.

Expression patterns of selected P450s genes from FAW larvae midguts of both Indox-SEL and Indox-UNSEL populations were analyzed by qRT-PCR (Figure 2). The 14 selected genes from clade-3 were; *CYP321A9*, *CYP6AN4*, *CYP6AE43*, *CYP337B5*, *CYP9A59*, *CYP321A7*, *CYP6AB12*, *CYP321B1*, *CYP321A10*, *CYP6B50*, *CYP321A8*, *CYP340L1*, *CYP321A9* and *CYP6AN4*. The eight selected genes from clade-4 were; *CYP4L4*, *CYP4C3*, *CYP4G74*, *CYP4G108*, *CYP4CG16*, *CYP321B1*, *CYP366A1*, and *CYP341A11*. The six selected genes from clade-2 were; *CYP306A1*, *CYP307A1*, *CYP18A1*, *CYP305A1*, *CYP301B1* and *CYP15C1*. In addition, 5 genes from the mitochondrial clade were selected; *CYP302A1*, *CYP49A1*, *CYP314A1*, *CYP315A1* and *CYP12B1*. It was shown that some cytochrome P450 genes from the four clades displayed significantly different expression patterns in the Indox-SEL population compared to the Indox-UNSEL population. Significantly higher mRNA transcript levels of two P450 genes, *CYP321A7* and *CYP6AE43*, from clade-3 were detected (11.22 and 9.07) in the midgut of Indox-SEL compared to the UNSEL population (Figure 2). Similarly, the highest expression level of *CYP6AE43* and *CYP321A7* was observed in the midguts of the larvae compared to the other tissues (Figure 3A). Meanwhile, significantly higher relative expression level of *CYP321A7* genes was detected in wings of adults as compared to other tissues (Figure 3B).

**FIGURE 4:**
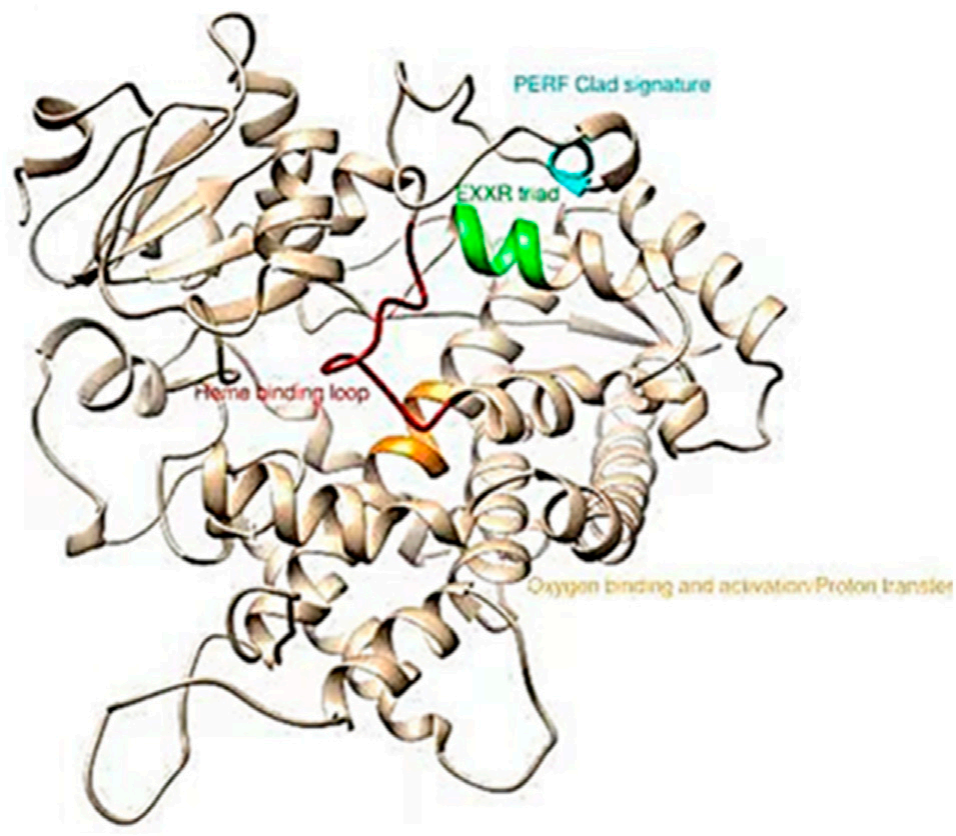
Conserved motifs in *S. frugiperda CYP6AE43* and *CYP321A7*. The protein sequences were queried against the pdb database to find template in ncbi blast.

MEGA 7 was used to perform phylogenetic analysis with the Minimum-Evolution method based on the amino acid sequences of all selected P450 genes belonging to CYP3, CYP2, CYP4 and the Mito-clade. The results showed that the mitochondrial clan and CYP4 appeared to share the maximum sequence similarity (Supplementary Figure S2). Amino acid alignments with different P450s of the *CYP6AE* and *CYP321A* subfamilies also indicated that the *CYP6AE43* and *CYP321A7* protein contains shared conserved motifs found in other P450s, including the helix C motif WKVQR (WxxxR), the helix I motif GFETS (Gx [ED]T [TS]), the helix K motif EALR (ExLR), the PERF motif PEQFRPER (PxxFxP [ED]RE) and the heme-binding motif PFGEGPRLCIG (PFxxGxRxCx [GA]) (Figure 4 and Supplementary Figure S1).

### The Integrity of Resulting dsRNAs

The integrity of all three resulting dsRNAs were analyzed by 1% agarose gel electrophoresis which showed the expected size of two P450 genes and control dsRED fragments as 679, 506 and 421 bp respectively (Supplementary Figure S3A and Supplementary Figure S3B).

### RNAi-mediated downregulation of ds*CYP321A7* and ds*CYP6AE43* genes in *S. frugiperda* via qRT-PCR

**FIGURE 5:**
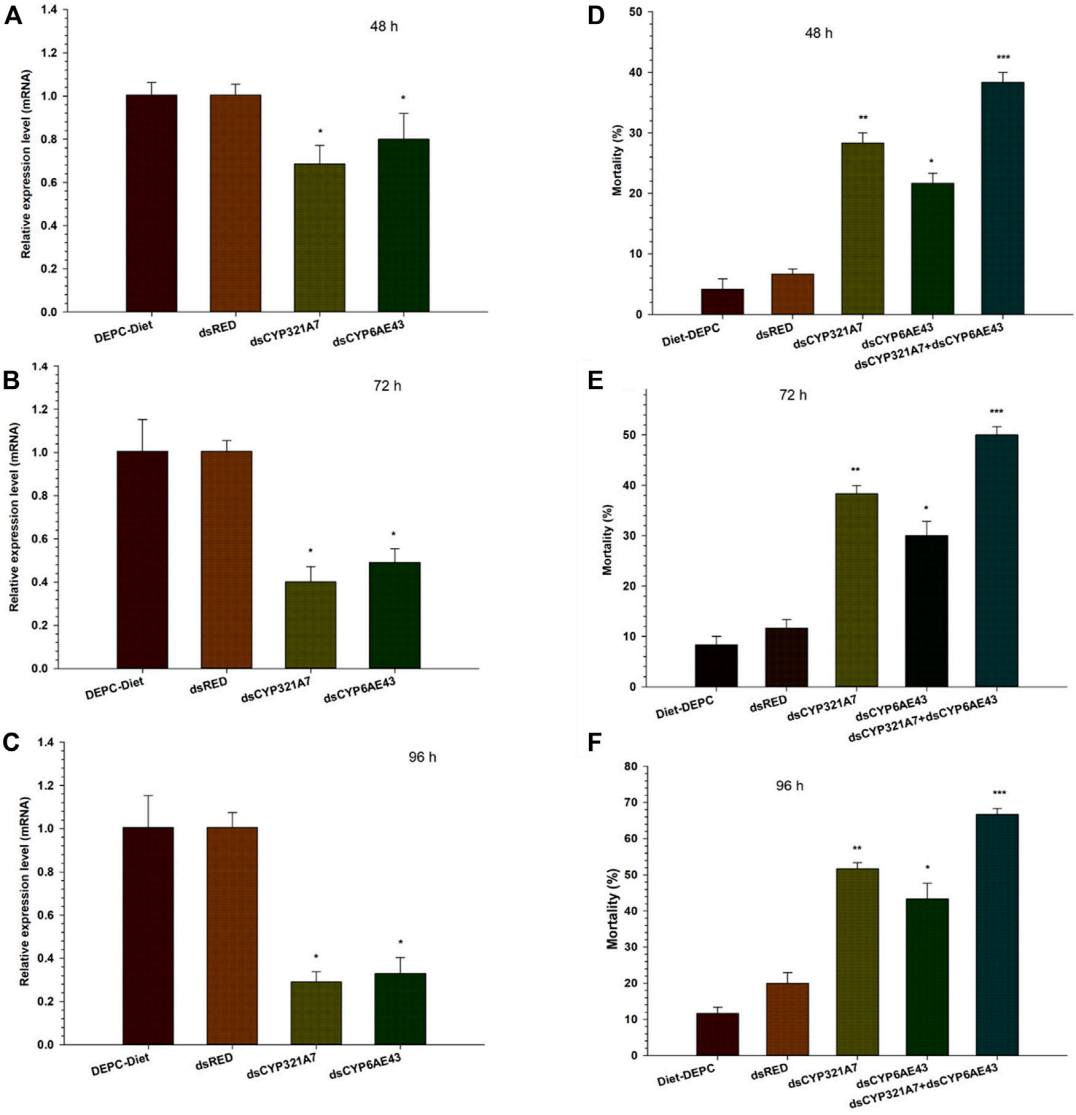
The relative mRNA transcript levels in the midguts of *S. frugiperda* larvae (Indox-SEL population) after feeding on dsCYP321A7, dsCYP6AE43 and dsRED for 48 h **(A)**, 72 h **(B)** and 96 h **(C)**. Similarly, the single dsCYP321A7, dsCYP6AE43 and combined dsCYP321A7+dsCYP6AE43) or dsRED effect of dsRNAs on the sensibility to indoxacarb in third-instar larvae of *S. frugiperda* after exposure with LC_20_: 6.84 μg g^−1^ concentration of indoxacarb. After feeding with dsRNAs and dsRED for 24 h followed by the exposed third instar larvae were transferred individually into 12-oriface tissue culture plate containing artificial diets supplemented with LC_20_: 6.84 μg g^−1^ of indoxacarb for 48 h **(D)**, 72 and 96 h **(F)**. DEPC-Diet as a control (Indox-UNSEL population) without exposure to indoxacarb. The mortality data were recorded at 48, 72 and 96 h. Data shown are means ± SE derived from three biological replicates. The astriks on the bar represent significant differences (*p* < 0.05) using one-way ANOVA, followed by Tukey’s HSD multiple comparison tests.

Two target P450 genes were selected to evaluate their function in indoxacarb resistant FAW*.* To find the RNAi efficiency, qRT-PCR was conducted to check the relative change in mRNA expression of targeted genes in FAW larval fed on dsRNAs, *dsCYP321A7* and *dsCYP6AE43* along with the dsRED control for 48, 72 and 96 h (Figure 4A–C). Dramatically lower mRNA transcript levels of *dsCYP321A7* and *dsCYP6AE43* genes were observed in Indox-SEL after 48, 72 and 96 h compared with the *dsRED* control (Figure 4A–C). We evaluated the effect of two dsRNAs targeting *CYP321A7* and *CYP6AE43* on the mortality of the indoxacarb resistant FAW larvae at different time points after feeding on dsRNA-supplemented diet followed by exposure to a lethal concentration (21.02 μg g^−1^) of indoxacarb (Figure 5D–F). After 48 h, a significantly higher mortality rate was observed in the *dsCYP321A7* and the *dsCYP6AE43*-fed larvae compared to the *dsRED* control (Figure 5D). The highest mortality was observed for the *dsCYP321A7* treatment. Feeding on a *dsCYP321A7*+*dsCYP6AE43*-supplemented diet significantly increased the mortality rate compared to all other treatments (Figure 5D). Similarly, a significantly higher mortality rate was observed in *dsCYP321A7* and *dsCYP6AE43*-fed larvae after 72 h compared to the *dsRED* control (Figure 5E). At 72 h, as previously, the highest mortality was observed in the combined treatment (Figure 5E). Furthermore, this trend was observed in the mortality rate recorded after 96 h (Figure 5F). The overall mortality rate was significantly increased in *dsCYP321A7*-fed larvae and, to a lesser extent, in *dsCYP6AE43*-fed larvae compared to the *dsRED* control (Figure 5F). However, the greatest mortality was observed when a combined treatment of *dsCYP321A7+dsCYP6AE43* supplemented diet was used (Figure 5F).

### The single and combined effect of dsRNA on the larval development, pupal development, and midgut physiology of FAW

**FIGURE 6:**
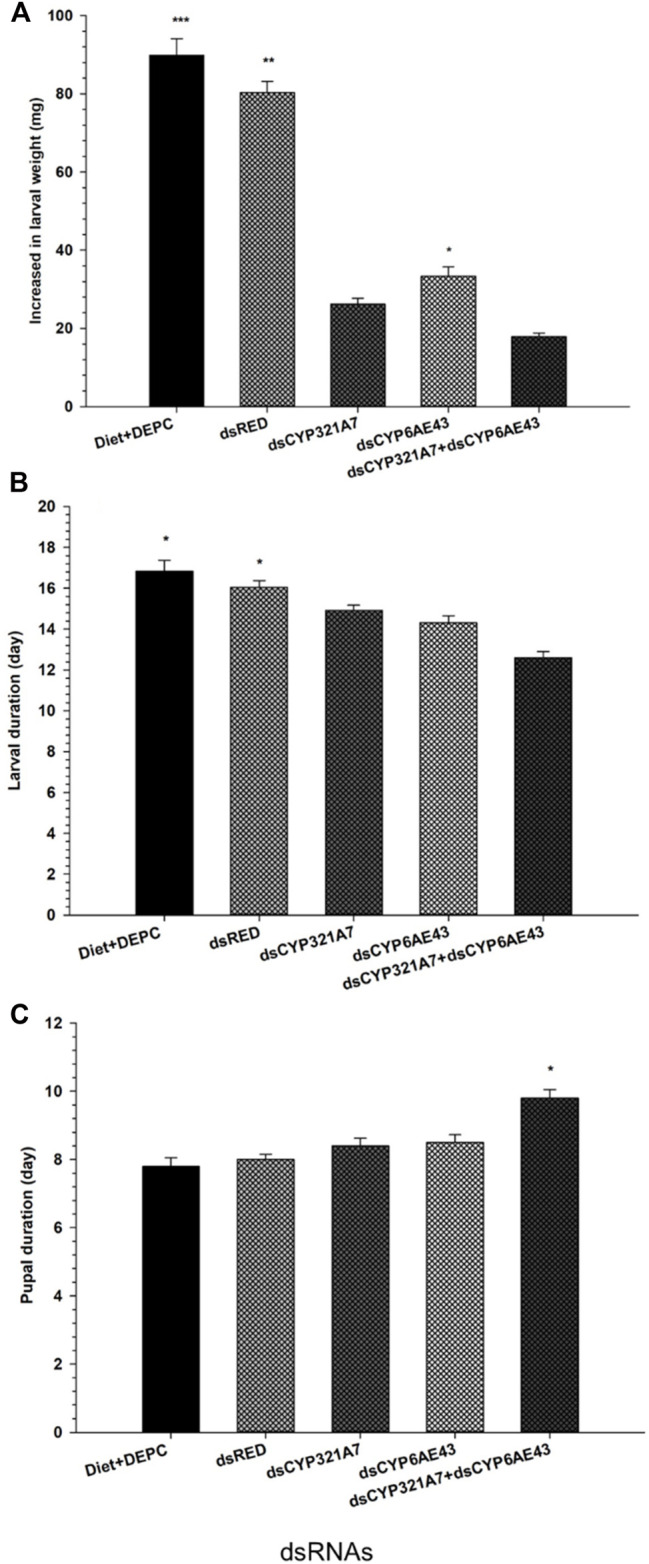
Single and combined effect of dsCYP321A7, dsCYP6AE43 and dsCYP321A7+dsCYP6AE43) or dsRED on larval duration **(A)**, increased in larval weight **(B)** pupal duration **(C)** of Indox-SEL population after feeding on dsRNAs, or the dsRED for 24 h followed by the exposed larvae were transferred individually into 12-oriface tissue culture plate containing artificial diets supplemented with LC_20_: 6.84 μg g^−1^ of indoxacarb for 72 h. DEPC-Diet as a control (Indox-UNSEL population) without exposure to indoxacarb. Data shown are means ± SE derived from three biological replicates. The astriks on the bar represent significant differences (*p* < 0.05) using one-way ANOVA, followed by Tukey’s HSD multiple comparison tests.

The larval developmental period of FAW in different treatment groups was assessed. Larvae were fed with dsRNA-supplemented diets targeting *dsCYP321A7* and *dsCYP6AE43*, or a combination of both, followed by exposure of LC_20_ (6.84 μg g^−1^) of indoxacarb after 3 days (Figure 6). It was found that these single target dsRNA-supplemented diets of *dsCYP321A7*, *dsCYP6AE43* and combined target dsRNA-supplemented diets of *dsCYP321A7*+*dsCYP6AE43* significantly reduced the larval duration in the Indox-SEL population compared to the *dsRED* and DEPC-water as control treatments (Figure 6A). While no significant decrease in larval duration between *dsCYP321A7* and *dsCYP6AE43* treatments was found (Figure 6A). Weight gain of the FAW larvae was found to be greatest in the control diet followed by the *dsRED* control group (Figure 6B). A significant reduction of the larvae weight gain was observed when the Indox-SEL population was fed on dsRNA-supplemented diet targeting *dsCYP321A7*, *dsCYP6AE43,* or a combination of both for 24 h followed by the exposure to LC_20_ (6.84 μg g^−1^) of indoxacarb after 3 days (Figure 6B). Furthermore, it was found that these single effects of *dsCYP321A7* and *dsCYP6AE43* did not affect the pupal duration, meanwhile pupal duration significantly increased when a combined *dsCYP321A7+dsCYP6AE43* treatment was applied in the Indox-SEL population compared to the *dsRED* and DEPC-water control treatments (Figure 6C).

**FIGURE 7:**
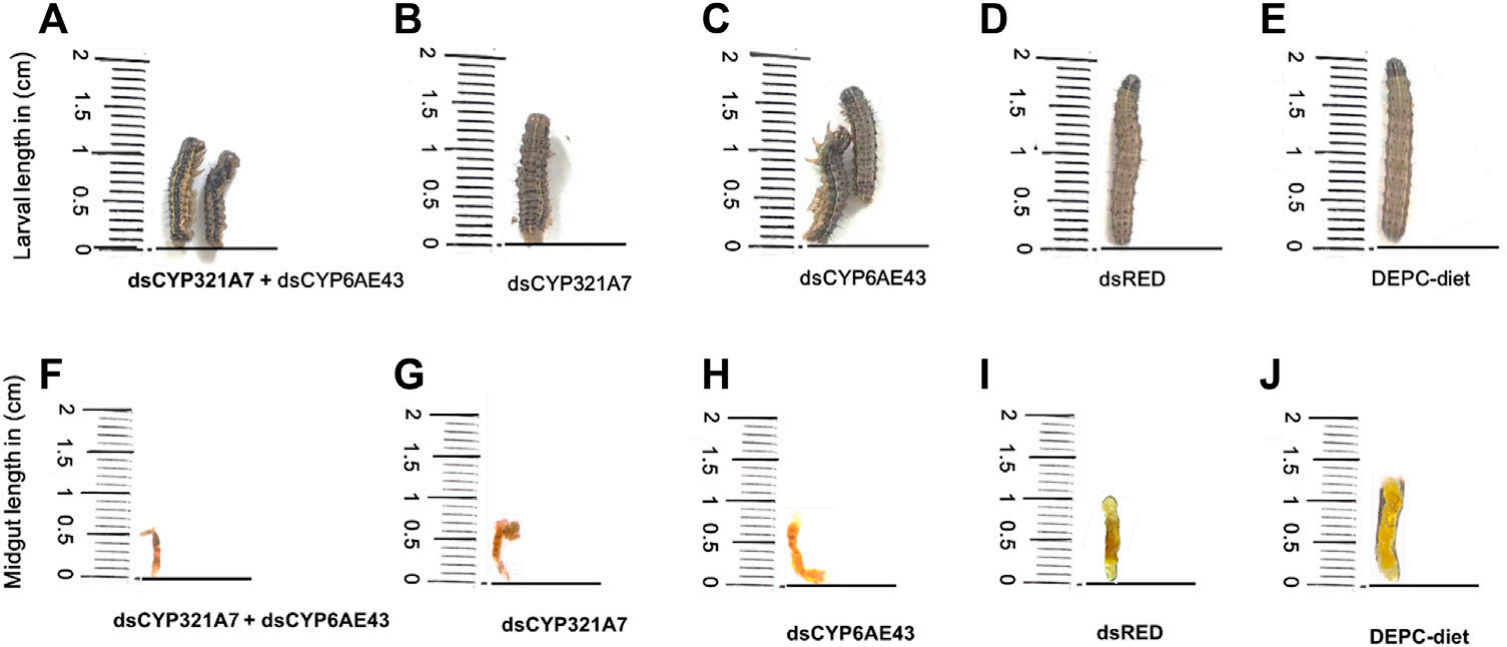
The effect of dsCYP321A7+dsCYP6AE43, dsCYP321A7, dsCYP6AE43, dsRED on the *S. frugiperda* larvae physiology and growth (Indox-SEL population) after feeding on dsRNAs, or the dsRED as a control for 24 h followed by exposure to diet supplemented with LC_20_: 6.84 μg g^−1^ concentration of indoxacarb for 72 h **(A,B,C,D)**. DEPC-Diet as a control (Indox-UNSEL population) without exposure to indoxacarb **(E)**. Similarly, the effect of dsCYP321A7+dsCYP6AE43, dsCYP321A7, dsCYP6AE43 and dsRED on the midguts physiology of *S. frugiperda* larvae (Indox-SEL population) after feeding on dsRNAs, or the dsRED as a control for 24 h followed by exposure to diet supplemented with LC_20_: 6.84 μg g^−1^ concentration of indoxacarb for 72 h **(F,G,H,I)**. DEPC-Diet as a control (Indox-UNSEL population) without exposure to indoxacarb **(J)**.

The changes in the larval and midguts physiology of the FAW after feeding on ds*CYP321A7* + *dsCYP6AE43*, *dsCYP321A7*, *dsCYP6AE43* for 24 h followed by the exposure to an artificial diet supplemented with LC_20_ (6.84 μg g^−1^) of indoxacarb for 72 h was assessed (Figure 7A–E). After exposure to dsRNAs for 24 h followed by LC_20_ (6.84 μg g^−1^) of indoxacarb insecticide, toxic symptoms including reduced appetite, shorter body length of larvae, the development and growth of most FAW were delayed. Furthermore, damaged midguts can be observed in single and combined dsRNAs-treated groups compared to the *dsRED* and DEPC-water control treatments (Figure 7F–J).

## Discussion

Synthetic insecticides are still the mainstay of insect pest management. Synthetic insecticides, including indoxacarb, have been extensively used against a variety of agricultural insect pests (Zhao et al., 2020), however, due to the development of insecticide resistance, these synthetic insecticides often fail to provide effective control (Gutirrez-Moreno et al., 2019; Boaventura et al., 2020; Zhang et al., 2020). The fall armyworm (FAW) has developed resistance to a range of different groups of synthetic insecticides including pyrethroids. An example is the now commonly observed resistance of FAW to lambda-cyhalothrin (Zhao et al., 2020).

To understand more about the important phenomena of indoxacarb resistance, we looked at the effect of silencing two important encoding P450 genes in FAW: *CYP321A7* and *CYP6AE43*. Our results indicated that PBO and TPP enhanced the toxicity of indoxacarb for the indoxacarb-resistant laboratory selected population (Indox-SEL). This indicates that cytochrome P450 monooxygenases may play a significant role in the resistance mechanism, because PBO as a synergist, can also block the non-specific esterase activity (Moores et al., 2009). It has been previously been identified that synthetic insecticides are converted into non-toxic compounds due to increased metabolic activity of detoxifying enzymes and reduced target sensitivity of pesticides, which is one of the key detoxification mechanisms in insect pests (Scott, 1999b; Feyereisen, 2006b; Li et al., 2007). We found that the enzyme activity of P450s monooxygenase in the Indox-SEL population was significantly enhanced after 24 h and up to 96 h following the exposure to indoxacarb. This pattern is similar to previous studies which have shown the greater detoxification activity of P450 enzymes in resistant populations of other insect pests (Chen et al., 2018; Wang et al., 2018c, 2018b). In addition to the involvement of P450s in resistance, the effects of esterase and GST contributing to indoxacarb resistance of FAW larvae should be further examined in future work.

The elevated activity of P450 enzymes due to the up-regulation of P450 genes is ostensibly a major mechanism for insecticide resistance (Elzaki et al., 2017; Ullah et al., 2020b). In the current study, we evaluated clades-3, 4, 2 and the mitochondrial clade P450 genes and found that two genes *CYP321A7* and *CYP6AE43* from clade CYP3 were associated with indoxacarb-resistant populations of FAW. Two P450 genes out of ten showed significantly higher expression levels in the midgut of FAW larvae after 14 generations of selection with indoxacarb. These findings were consistent with previous findings that some of the *CYP321* subfamily genes, as well as the CYP6 and CYP9 subfamily genes, may be involved in mechanisms of detoxication or regulation of insecticides. These findings elucidated P450 functions on insect biology and physiology, and that the midgut and fat body tissue of insects are considered important as detoxification organs (Giraudo et al., 2015; Hou et al., 2021). It has been previously reported that the development of insecticide resistance by enhancing metabolic detoxification enzymes in various insects is closely related to the elevated expression of P450 genes and subsequent increases in P450 protein levels (Riveron et al., 2013; Xu et al., 2018). In this study, we tested whether the resistant populations of *S. frugiperda* differ in expression of P450 genes of CYP4, CYP6 and CYP9 subfamilies, which are expected to be associated with insecticide resistance (Bai-Zhong et al., 2020; Wang et al., 2022; Zhao et al., 2022). Among the differentially expressed *S. frugiperda* P450 genes identified in this study, CYP321A7 and CYP6AE43 genes were particularly overexpressed in larvae of indoxacarb-resistant populations (11.6 and 9.4 fold higher in resistant compared to control populations). These results are in line with previous findings showing that the insecticide-induced P450 genes (*CYP6BG1, CYP321A8, CYP321B1, CYP9A32, CYP333B3, CYP9A26, CYP321A9*, *CYP337B5*, and *CYP6AE44*) that play a role in insect metabolic resistance (Bautista et al., 2009; Giraudo et al., 2015; Bai-Zhong et al., 2020). Similarly, transcriptome analysis has revealed higher mRNA expression level of five P450 genes in indoxacarb-fed larvae of *Spodoptera exigua* (Hu et al., 2019)*.* The induced mRNA expression of some P450 genes, *CYP9A9*, *CYP321A1* and *CYP321B1* has been reported in the midgut of FAW and *Spodoptera litura* after exposure to different insecticides (Nascimento et al., 2015; Wang et al., 2017). Taken together with the present results, this indicated that indoxacarb resistance in FAW is a more complex mechanism than initially considered. The phylogenetic tree (Supplementary Figure S3A) demonstrates that *CYP6AE43* and *CYP321A7* belong to the *CYP6AE* subfamily (*CYP3* clan of insect P450s). P450s from the CYP6AE subfamily of insect P450s have been shown in a number of studies to play an important role in the development of insecticide resistance (Hu et al., 2017; Shi et al., 2018), this study was focused on *CYP6AE43* and *CYP321A7*. The amino acid sequences of *CYP6AE43* and *CYP321A7* contain various shared conserved motifs including heme-binding predicted substrate recognition sites, suggesting that the enzyme is functional. These results are in line with previous findings (Guo et al., 2015; Hou et al., 2021).

To further explore if the indoxacarb-induced genes *CYP321A7* and *CYP6AE43* are involved in indoxacarb detoxification, we fed specific *dsCYP321A7* and *dsCYP6AE43 S. fugiperda* larvae to silence target genes and then analyzed phenotypic effects. Greater larval mortality was observed in the resistant populations of FAW after feeding on dsRNAs-fed (*CYP321A7* and *CYP6AE43*) followed by the exposure of an LC_20_ concentration of indoxacarb as compared to dsRED and Diet-DEPC as a control (Figure 5A–C). Similar findings have been seen previously by Naqqash *et al.* (2020) and Bai-Zhong *et al.* (2020) who reported that the subfamilies of the CYP321, CYP6, and CYP9 of P450 genes could be induced by insecticides. Similarly, we found that downregulation of P450 genes significantly reduced the larval developmental time, larval growth and larval weight following feeding on the dsRNAs-supplemented diet with subsequent indoxacarb exposure. Naqqash *et al.* (2020) and Zhao *et al.* (2016) also documented that the larval growth and development was significantly reduced in *H. armigera* and *Leptinotarsa decemlineata* due to dsRNAs feeding. Studies continue to reveal the dynamic role P450s play in the regulation of growth and the development of insecticide resistance in various insects. Therefore, it is understood that the downregulation of these genes via RNAi could result in higher mortality across various insect species (Zhang et al., 2013; Jin et al., 2015; Zhao et al., 2016; Bai-Zhong et al., 2020; Hafeez et al., 2020). Knocking down of P450 genes increased larval mortality, reduced developmental duration, and increased insecticide susceptibility, indicating the role of P450 genes in larval growth, development, and sensitivity to indoxacarb. Physiological and biochemical studies have shown that P450 enzymes are vital to in insect hormone metabolism pathways but details of the molecular processes remain unknown (Iga and Kataoka, 2012). The growth of FAW larvae was hindered after downregulation of *CYP321A7* and *CYP6AE43* by RNAi, but how these P450 genes regulate this process requires further study.

## Conclusion

It was shown that the droplet feeding of dsRNAs (*CYP321A7* and *CYP6AE43*) via artificial diet significantly increased mortality rates of indoxacarb-resistant larvae. Shorter larval developmental time was detected in the dsRNAs-exposed larvae of FAW. Similarly, larval weight was also reduced in the dsRNAs-exposed resistant population. To our knowledge, this is the first time an indoxacarb-resistant FAW population was used to evaluate the effects of different dsRNA on larval mortality, growth, and insecticide susceptibility. Our results elucidate some of the effects of the RNAi technique and the function of specific genes. The application of dsRNA may offer a route to reduce the development of resistance in FAW against various insecticides in the field. This paves the route for further study into the suppression of resistance in fall armyworm and various other important agricultural insect pests.

## Data Availability

The original contributions presented in the study are included in the article/Supplementary Material, further inquiries can be directed to the corresponding authors.

## References

[B2] Ahmad M. Sayyed A. H. Saleem M. A. Ahmad M. (2008). Evidence for Field Evolved Resistance to Newer Insecticides in *Spodoptera Litura* (Lepidoptera: Noctuidae) from Pakistan. Crop Prot. 27, 1367–1372. 10.1016/j.cropro.2008.05.003

[B3] Aktar W. Sengupta D. Chowdhury A. (2009). Impact of Pesticides Use in Agriculture: Their Benefits and Hazards. Interdiscip. Toxicol. 2, 1–12. 10.2478/v10102-009-0001-7 21217838 PMC2984095

[B4] Alves A. P. Allgeier W. J. Siegfried B. D. (2008). Effects of the Synergist S,S,S-tributyl Phosphorotrithioate on Indoxacarb Toxicity and Metabolism in the European Corn Borer, Ostrinia Nubilalis (Hübner). Pestic. Biochem. Physiol. 90, 26–30. 10.1016/j.pestbp.2007.07.005

[B5] Arain M. S. Shakeel M. Elzaki M. E. A. Farooq M. Hafeez M. Shahid M. R. (2018). Association of Detoxification Enzymes with Butene-Fipronil in Larvae and Adults of *Drosophila melanogaster* . Environ. Sci. Pollut. Res. 25, 10006–10013. 10.1007/s11356-018-1202-4 29380196

[B6] Bai-Zhong Z. Xu S. Cong-Ai Z. Liu-Yang L. Ya-She L. Xing G. (2020). Silencing of Cytochrome P450 in *Spodoptera Frugiperda* (Lepidoptera: Noctuidae) by RNA Interference Enhances Susceptibility to Chlorantraniliprole. J. Insect Sci. 20, 1–7. 10.1093/jisesa/ieaa047 PMC7266073 32484869

[B7] Bautista M. A. M. Miyata T. Miura K. Tanaka T. (2009). RNA Interference-Mediated Knockdown of a Cytochrome P450, CYP6BG1, from the Diamondback Moth, *Plutella Xylostella*, Reduces Larval Resistance to Permethrin. Insect Biochem. Mol. Biol. 39, 38–46. 10.1016/j.ibmb.2008.09.005 18957322

[B8] Bennett M. Deikman J. Hendrix B. Iandolino A. (2020). Barriers to Efficient Foliar Uptake of dsRNA and Molecular Barriers to dsRNA Activity in Plant Cells. Front. Plant Sci. 11, 816. 10.3389/fpls.2020.00816 32595687 PMC7304407

[B9] Boaventura D. Bolzan A. Padovez F. E. Okuma D. M. Omoto C. Nauen R. (2020). Detection of a Ryanodine Receptor Target‐site Mutation in Diamide Insecticide Resistant Fall Armyworm, Spodoptera Frugiperda. Pest Manag. Sci. 76, 47–54. 10.1002/ps.5505 31157506

[B10] Bradford M. M. (1976). A Rapid and Sensitive Method for the Quantitation of Microgram Quantities of Protein Utilizing the Principle of Protein-Dye Binding. Anal. Biochem. 72, 248–254. 10.1016/0003-2697(76)90527-3 942051

[B11] Burtet L. M. Bernardi O. Melo A. A. Pes M. P. Strahl T. T. Guedes J. V. (2017). Managing Fall Armyworm, *Spodoptera Frugiperda* (Lepidoptera: Noctuidae), with Bt maize and Insecticides in Southern Brazil. Pest Manag. Sci. 73, 2569–2577. 10.1002/ps.4660 28695664

[B12] Bustin S. A. Benes V. Garson J. A. Hellemans J. Huggett J. Kubista M. (2009). The MIQE Guidelines: Minimum Information for Publication of Quantitative Real-Time PCR Experiments. Clin. Chem. 55 (4), 611–622. 10.1373/clinchem.2008.112797 19246619

[B13] Chen C. Han P. Yan W. Wang S. Shi X. Zhou X. (2018). Uptake of Quercetin Reduces Larval Sensitivity to Lambda-Cyhalothrin in *Helicoverpa Armigera* . J. Pest Sci. 91, 919–926. 10.1007/s10340-017-0933-1

[B14] Chen X. Head G. P. Price P. Kerns D. L. Rice M. E. Huang F. (2019). Fitness Costs of Vip3A Resistance in *Spodoptera Frugiperda* on Different Hosts. Pest Manag. Sci. 75, 1074–1080. 10.1002/ps.5218 30242959

[B15] Cordova D. Benner E. A. Sacher M. D. Rauh J. J. Sopa J. S. Lahm G. P. (2006). Anthranilic Diamides: A New Class of Insecticides with a Novel Mode of Action, Ryanodine Receptor Activation. Pestic. Biochem. Physiol. 84, 196–214. 10.1016/j.pestbp.2005.07.005

[B16] Davis C. C. (1993). Environmental Concerns about Pesticide Use in Philippine Agriculture. Sci. Total Environ. 134, 293–306. 10.1016/S0048-9697(05)80030-0

[B1] Day R. Abrahams P. Bateman M. Beale T. Clottey V. Cock M. (2017). Fall Armyworm: Impacts and Implications for Africa. Outlooks Pest Manag 28, 196–201. 10.1564/v28_oct_02

[B90] Desneux N. Decourtye A. Delpuech J. (2007). The Sublethal Effects of Pesticides on Beneficial Arthropods.. Annu Rev Entomol 52:81 52, 81–106. 10.1146/annurev.ento.52.110405.091440 16842032

[B17] Ehrlich P. R. Raven P. H. (1964). Butterflies and Plants: A Study in Coevolution. Evolution 18, 586–608. 10.2307/240621210.1111/j.1558-5646.1964.tb01674.x

[B18] Elzaki M. Miah M. Han Z. (2017). Buprofezin Is Metabolized by CYP353D1v2, a Cytochrome P450 Associated with Imidacloprid Resistance in *Laodelphax Striatellus* . Ijms 18, 2564. 10.3390/ijms18122564 PMC5751167 29186030

[B19] Feyereisen R. (2006b). Evolution of Insect P450. Biochem. Soc. Trans. 34, 1252–1255. 10.1042/BST0341252 17073796

[B20] Feyereisen R. (2006a). Evolution of Insect P450. Biochem. Soc. Trans. 34, 1252–1255. 10.1042/BST0341252 17073796

[B21] Feyereisen R. (2012). Insect CYP Genes and P450 Enzymes. Insect Mol. Biol. Biochem., 236–316. 10.1016/B978-0-12-384747-8.10008-X

[B22] Ginting S. Zarkani A. Hadi Wibowo R. (2020). New invasive pest, *Spodoptera frugiperda* (J. e. smith) (lepidoptera: Noctuidae) attacking corn in bengkulu. indonesia: Serangga 25, 105–117.

[B23] Giraudo M. Hilliou F. Fricaux T. Audant P. Feyereisen R. Le Goff G. (2015). Cytochrome P450s from the Fall Armyworm (*Spodoptera Frugiperda*): Responses to Plant Allelochemicals and Pesticides. Insect Mol. Biol. 24, 115–128. 10.1111/imb.12140 25315858

[B24] Goergen G. Kumar P. L. Sankung S. B. Togola A. Tamò M. (2016). First Report of Outbreaks of the Fall Armyworm *Spodoptera Frugiperda* (J E Smith) (Lepidoptera, Noctuidae), a New Alien Invasive Pest in West and Central Africa. PLoS One 11, e0165632. 10.1371/journal.pone.0165632 27788251 PMC5082806

[B89] Gul H. Ullah F. Hafeez M. (2021). Sublethal Concentrations of Clothianidin Affect Fecundity and Key Demographic Parameters of the Chive Maggot, Bradysia Odoriphaga.. Ecotoxicology 30, 1150–1160. 10.1007/s1064602102446x 34165677

[B25] Guo Y. Zhang X. Wu H. Yu R. Zhang J. Zhu K. Y. (2015). Identification and Functional Analysis of a Cytochrome P450 Gene CYP9AQ2 Involved in Deltamethrin Detoxification from *Locusta migratoria* . Pestic. Biochem. Physiol. 122, 1–7. 10.1016/j.pestbp.2015.01.003 26071800

[B26] Gupta G. P. Rani S. Birah A. Raghuraman M. (2005). Improved Artificial Diet for Mass Rearing of the Tobacco Caterpillar, *Spodoptera Litura* (Lepidoptera: Noctuidae). Int. J. Tropical. Insect. Sci. 25, 55–58. 10.1079/IJT200551

[B27] Gutiérrez-Moreno R. Mota-Sanchez D. Blanco C. A. Whalon M. E. Terán-Santofimio H. Rodriguez-Maciel J. C. (2019). Field-Evolved Resistance of the Fall Armyworm (Lepidoptera: Noctuidae) to Synthetic Insecticides in Puerto Rico and Mexico. J. Econ. Entomol. 112, 792–802. 10.1093/jee/toy372 30535077

[B28] Hafeez M. Liu S. Jan S. Ali B. Shahid M. Fernández-Grandon G. M. (2019a). Gossypol-induced Fitness Gain and Increased Resistance to Deltamethrin in Beet armyworm,Spodoptera exigua(Hübner). Pest Manag. Sci. 75, 683–693. 10.1002/ps.5165 30094908

[B29] Hafeez M. Liu S. Jan S. Gulzar A. Fernández-Grandon G. M. Qasim M. (2019b). Enhanced Effects of Dietary Tannic Acid with Chlorantraniliprole on Life Table Parameters and Nutritional Physiology of Spodoptera Exigua (Hübner). Pestic. Biochem. Physiol. 155, 108–118. 10.1016/j.pestbp.2019.01.012 30857620

[B30] Hafeez M. Liu S. Jan S. Shi L. Fernández-Grandon G. M. Gulzar A. (2019c). Knock-Down of Gossypol-Inducing Cytochrome P450 Genes Reduced Deltamethrin Sensitivity in Spodoptera Exigua (Hübner). Ijms 20, 2248. 10.3390/ijms20092248 PMC6539524 31067723

[B92] Hafeez M. Jan S. Nawaz M. (2019d). Sub Lethal Effects of Lufenuron Exposure on Spotted Bollworm Earias Vittella (Fab): Key Biological Traits and Detoxification Enzymes Activity. Environ Sci Pollut Res 26, 14300–14312. 10.1007/s11356019046558 30864030

[B93] Hafeez M. Liu S. Jan S. (2019e). Gossypol-Induced Fitness Gain and Increased Resistance to Deltamethrin in Beet Armyworm, Spodoptera Exigua (Hübner). Pest Manag Sci 76, 683–693. 10.1002/ps.5165 30094908

[B31] Hafeez M. Liu S. Yousaf H. K. Jan S. Wang R.-L. Fernández-Grandon G. M. (2020). RNA Interference-Mediated Knockdown of a Cytochrome P450 Gene Enhanced the Toxicity of α-cypermethrin in Xanthotoxin-Fed Larvae of Spodoptera Exigua (Hübner). Pestic. Biochem. Physiol. 162, 6–14. 10.1016/j.pestbp.2019.07.003 31836055

[B32] Hafez A. M. Mota-Sanchez D. Hollingworth R. M. Vandervoort C. Wise J. C. (2020). Metabolic Mechanisms of Indoxacarb Resistance in Field Populations of *Choristoneura Rosaceana* (Harris) (Lepidoptera: Tortricidae). Pestic. Biochem. Physiol. 168, 104636. 10.1016/j.pestbp.2020.104636 32711770

[B33] Hou W.-T. Staehelin C. Elzaki M. E. A. Hafeez M. Luo Y.-S. Wang R.-L. (2021). Functional Analysis of CYP6AE68, a Cytochrome P450 Gene Associated with Indoxacarb Resistance in *Spodoptera Litura* (Lepidoptera: Noctuidae). Pestic. Biochem. Physiol. 178, 104946. 10.1016/j.pestbp.2021.10494610.1016/j.pestbp.2021.104946 34446184

[B34] Hu B. Zhang S. H. Ren M. M. Tian X. R. Wei Q. Mburu D. K. (2017). The Expression of *Spodoptera Exigua* P450 and UGT Genes: Tissue Specificity and Response to Insecticides. Insect Sci. 26, 199–216. 10.1111/1744-7917.12538 28881445 PMC7379962

[B35] Hu B. Zhang S. H. Ren M. M. Tian X. R. Wei Q. Mburu D. K. (2019). The Expression of *Spodoptera Exigua* P450 and UGT Genes: Tissue Specificity and Response to Insecticides. Insect Sci. 26, 199–216. 10.1111/1744-7917.12538 28881445 PMC7379962

[B36] Iftikhar A. Hafeez F. Hafeez M. Farooq M. Asif Aziz M. Sohaib M. (2020). Sublethal Effects of a Juvenile Hormone Analog, Pyriproxyfen on Demographic Parameters of Non-target Predator, *Hippodamia convergens* Guerin-Meneville (Coleoptera: Coccinellidae). Ecotoxicology 29, 1017–1028. 10.1007/s10646-020-02159-7 31955283

[B37] Iga M. Kataoka H. (2012). Recent Studies on Insect Hormone Metabolic Pathways Mediated by Cytochrome P450 Enzymes. Biol. Pharm. Bull. 35, 838–843. 10.1248/bpb.35.838 22687472

[B38] Jan S. Liu S. Hafeez M. Zhang X. Dawar F. U. Guo J. (2017). Isolation and Functional Identification of Three Cuticle Protein Genes during Metamorphosis of the Beet Armyworm, Spodoptera Exigua. Sci. Rep. 7, 16061. 10.1038/s41598-017-16435-w 29167522 PMC5700046

[B39] Jin S. Singh N. D. Li L. Zhang X. Daniell H. (2015). Engineered Chloroplast dsRNA Silences Cytochrome P450 Monooxygenase , V ‐ ATPase and Chitin Synthase Genes in the Insect Gut and Disrupts Helicoverpa Armigera Larval Development and Pupation. Plant Biotechnol. J. 13, 435–446. 10.1111/pbi.12355 25782349 PMC4522700

[B40] Li T. Liu N. (2017). Regulation of P450-Mediated Permethrin Resistance in *Culex quinquefasciatus* by the GPCR/Gαs/AC/cAMP/PKA Signaling cascade. Biochem. Biophys. Rep. 12, 12–19. 10.1016/j.bbrep.2017.08.010 28955787 PMC5613228

[B41] Li X. J. Wu M. F. Ma J. Gao B. Y. Wu Q. L. Chen A. D. (2020). Prediction of Migratory Routes of the Invasive Fall Armyworm in Eastern China Using a Trajectory Analytical Approach. Pest Manag. Sci. 76, 454–463. 10.1002/ps.5530 31237729

[B42] Li X. Schuler M. A. Berenbaum M. R. (2007). Molecular Mechanisms of Metabolic Resistance to Synthetic and Natural Xenobiotics. Annu. Rev. Entomol. 52, 231–253. 10.1146/annurev.ento.51.110104.151104 16925478

[B43] Lim Z. X. Robinson K. E. Jain R. G. Sharath Chandra G. Asokan R. Asgari S. (2016). Diet-delivered RNAi in *Helicoverpa Armigera* - Progresses and Challenges. J. Insect Physiol. 85, 86–93. 10.1016/j.jinsphys.2015.11.005 26549127

[B44] Liu X. N. Liang P. Gao X. W. Shi X. Y. (2006). Induction of the Cytochrome P450 Activity by Plant Allelochemicals in the Cotton Bollworm, *Helicoverpa Armigera* (Hubner). Pestic. Biochem. Physiol. 84, 127–134.

[B45] Liu Y. Qi M. Chi Y. Wuriyanghan H. (2016). De Novo Assembly of the Transcriptome for Oriental ArmywormMythimna separata(Lepidoptera: Noctuidae) and Analysis on Insecticide Resistance-Related Genes. J. Insect Sci. 16, 92. 10.1093/jisesa/iew079 27638951 PMC5026479

[B46] Livak K. J. Schmittgen T. D. (2001). Analysis of Relative Gene Expression Data Using Real-Time Quantitative PCR and the 2−ΔΔCT Method. Methods 25, 402–408. 10.1006/meth.2001.1262 11846609

[B47] Lu K. Cheng Y. Li W. Li Y. Zeng R. Song Y. (2020). Activation of CncC Pathway by ROS Burst Regulates Cytochrome P450 CYP6AB12 Responsible for λ-cyhalothrin Tolerance in Spodoptera Litura. J. Hazard. Mater. 387, 121698. 10.1016/j.jhazmat.2019.121698 31791865

[B48] Mallet J. (1989). The Evolution of Insecticide Resistance: Have the Insects Won? Trends Ecol. Evol. 4, 336–340. 10.1016/0169-5347(89)90088-8 21227375

[B49] McCann S. F. Annis G. D. Shapiro R. Piotrowski D. W. Lahm G. P. Long J. K. (2001). The Discovery of Indoxacarb: Oxadiazines as a New Class of Pyrazoline-type Insecticides. Pest Manag. Sci. 57, 153–164. 10.1002/1526-4998(200102)57:2<153::aid-ps288>3.0.co;2-o 11455646

[B50] Mello C. C. Conte D. (2004). Revealing the World of RNA Interference. Nature 431, 338–342. 10.1038/nature02872 15372040

[B51] Monnerat R. Martins E. Macedo C. Queiroz P. Praça L. Soares C. M. (2015). Evidence of Field-Evolved Resistance of *Spodoptera Frugiperda* to Bt Corn Expressing Cry1F in Brazil that Is Still Sensitive to Modified Bt Toxins. PLoS One 10, e0119544. 10.1371/journal.pone.0119544 25830928 PMC4382162

[B52] Montezano D. G. Specht A. Sosa-Gómez D. R. Roque-Specht V. F. Sousa-Silva J. C. Paula-Moraes S. V. (2018). Host Plants ofSpodoptera frugiperda(Lepidoptera: Noctuidae) in the Americas. Afr. Entomol. 26, 286–300. 10.4001/003.026.0286

[B53] Moores G. D. Philippou D. Borzatta V. Trincia P. Jewess P. Gunning R. (2009). An Analogue of Piperonyl Butoxide Facilitates the Characterisation of Metabolic Resistance. Pest Manag. Sci. 65, 150–154. 10.1002/ps.1661 18951417

[B54] Naqqash M. N. Gökçe A. Aksoy E. Bakhsh A. (2020). Downregulation of Imidacloprid Resistant Genes Alters the Biological Parameters in Colorado Potato Beetle, *Leptinotarsa decemlineata* Say (Chrysomelidae: Coleoptera). Chemosphere 240, 124857. 10.1016/j.chemosphere.2019.124857 31726599

[B55] Nascimento A. R. B. d. Fresia P. Cônsoli F. L. Omoto C. (2015). Comparative Transcriptome Analysis of Lufenuron-Resistant and Susceptible Strains of *Spodoptera Frugiperda* (Lepidoptera: Noctuidae). BMC Genomics 16, 985. 10.1186/s12864-015-2183-z 26589731 PMC4654862

[B56] Nawaz M. Hafeez M. Mabubu J. I. Dawar F. U. Li X. Khan M. M. (2018). Transcriptomic Analysis of Differentially Expressed Genes and Related Pathways in *Harmonia axyridis* after Sulfoxaflor Exposure. Int. J. Biol. Macromolecules 119, 157–165. 10.1016/j.ijbiomac.2018.07.032 30009920

[B57] Nehare S. Moharil M. P. Ghodki B. S. Lande G. K. Bisane K. D. Thakare A. S. (2010). Biochemical Analysis and Synergistic Suppression of Indoxacarb Resistance in Plutella Xylostella L. J. Asia-Pacific Entomol. 13, 91–95. 10.1016/j.aspen.2009.12.002

[B58] Riveron J. M. Irving H. Ndula M. Barnes K. G. Ibrahim S. S. Paine M. J. I. (2013). Directionally Selected Cytochrome P450 Alleles Are Driving the Spread of Pyrethroid Resistance in the Major Malaria Vector Anopheles Funestus. Proc. Natl. Acad. Sci. U.S.A. 110, 252–257. 10.1073/pnas.1216705110 23248325 PMC3538203

[B59] Robertson J. L. Preisler H. K. (1992). Pesticide Bioassays with Arthropods. Boca Raton, FL: CRC Press, CRC.

[B60] Scott J. G. (1999a). Cytochromes P450 and Insecticide Resistance. Insect Biochem. Mol. Biol. 10.1016/S0965-1748(99)00038-7 10510498

[B61] Scott J. G. (1999b). Cytochromes P450 and Insecticide Resistance. Insect Biochem. Mol. Biol. 29, 757–777. 10.1016/S0965-1748(99)00038-7 10510498

[B62] Shi Y. Wang H. Liu Z. Wu S. Yang Y. Feyereisen R. (2018). Phylogenetic and Functional Characterization of Ten P450 Genes from the CYP6AE Subfamily of *Helicoverpa Armigera* Involved in Xenobiotic Metabolism. Insect Biochem. Mol. Biol. 93, 79–91. 10.1016/j.ibmb.2017.12.006 29258871

[B63] Sun Z. Shi Q. Li Q. Wang R. Xu C. Wang H. (2019). Identification of a Cytochrome P450 CYP6AB60 Gene Associated with Tolerance to Multi-Plant Allelochemicals from a Polyphagous Caterpillar Tobacco Cutworm (*Spodoptera Litura*). Pestic. Biochem. Physiol. 154, 60–66. 10.1016/j.pestbp.2018.12.006 30765057

[B64] Tang B. Cheng Y. Li Y. Li W. Ma Y. Zhou Q. (2020). Adipokinetic Hormone Enhances CarE ‐mediated Chlorpyrifos Resistance in the Brown Planthopper, Nilaparvata Lugens. Insect Mol. Biol. 29, 511–522. 10.1111/imb.1265910.1111/imb.12659 32686884

[B65] Thompson G. D. Dutton B. (2003). Insecticide Resistance Action Committee (IRAC). Pest Outlook 14, 146. 10.1039/b308501p

[B91] Ullah F. Gul H. Desneux N. (2019). Imidacloprid Induced Hormesis Effects on Demographic Traits of the Melon Aphid, Aphis Gossypii. Entomolgen 39, 325–337. 10.1127/entomologia/2019/0892

[B94] Ullah F. Gul H. Tariq K. Murtaza M. Ali A. Desneux N. (2021). Expression changes of cytochrome P450 genes at low lethal and sublethal concentrations of acetamiprid in melon aphid, Aphis gossypii January 2021. Conference: International Conference on Smart Plant Protection At: Institute of Plant Protection, MNS University of Agriculture, Multan SPP-IPM-104

[B95] Ullah F. Gul H. Tariq K. Desneux N. Gao X. Song D. (2020a). Functional analysis of cytochrome P450 genes linked with acetamiprid resistance in melon aphid, Aphis gossypii. Pestic. Biochem. Physiol 170, 104687. 10.1016/j.pestbp.2020.104687 32980055

[B96] Ullah F. Gul H. Yousaf H. K. Xiu W. Qian D. Gao X. (2020b). Author Correction: Impact of low lethal concentrations of buprofezin on biological traits and expression profile of chitin synthase 1 gene (CHS1) in melon aphid, Aphis gossypii. Sci. Rep. 9 (1), 12291. 10.1038/s41598-020-74318-z PMC6707215 31444364

[B66] van Asperen K. (1962). A Study of Housefly Esterases by Means of a Sensitive Colorimetric Method. J. Insect Physiol. 8, 401–416. 10.1016/0022-1910(62)90074-4

[B67] Vontas J. G. Small G. J. Hemingway J. (2000). Comparison of Esterase Gene Amplification, Gene Expression and Esterase Activity in Insecticide Susceptible and Resistant Strains of the Brown Planthopper, Nilaparvata Lugens (Stal). Insect Mol. Biol. 9, 655–660. 10.1046/j.1365-2583.2000.00228.x 11122475

[B68] Wang K. Zhao J. Han Z. Chen M. (2022). Comparative Transcriptome and RNA Interference Reveal CYP6DC1 and CYP380C47 Related to Lambda-Cyhalothrin Resistance in Rhopalosiphum Padi. Pestic. Biochem. Physiol. 183, 105088. 10.1016/j.pestbp.2022.105088 35430059

[B69] Wang R.-L. Zhu-Salzman K. Baerson S. R. Xin X.-W. Li J. Su Y.-J. (2017). Identification of a Novel Cytochrome P450 CYP321B1 Gene from Tobacco Cutworm (*Spodoptera Litura*) and RNA Interference to Evaluate its Role in Commonly Used Insecticides. Insect Sci. 24, 235–247. 10.1111/1744-7917.12315 26782704

[B70] Wang X. Chen Y. Gong C. Yao X. Jiang C. Yang Q. (2018a). Molecular Identification of Four Novel Cytochrome P450 Genes Related to the Development of Resistance of *Spodoptera Exigua* (Lepidoptera: Noctuidae) to Chlorantraniliprole. Pest Manag. Sci. 74, 1938–1952. 10.1002/ps.4898 29488686

[B71] Wang X. Huang Q. Hao Q. Ran S. Wu Y. Cui P. (2018b). Insecticide Resistance and Enhanced Cytochrome P450 Monooxygenase Activity in Field Populations of *Spodoptera Litura* from Sichuan, China. Crop Prot. 106, 110–116. 10.1016/j.cropro.2017.12.020

[B72] Wang X. Khakame S. K. Ye C. Yang Y. Wu Y. (2013). Characterisation of Field-Evolved Resistance to Chlorantraniliprole in the Diamondback moth,Plutella Xylostella, from China. Pest Manag. Sci. 69, 661–665. 10.1002/ps.3422 23109334

[B73] Wang X. Lou L. Su J. (2019). Prevalence and Stability of Insecticide Resistances in Field Population of Spodoptera Litura (Lepidoptera: Noctuidae) from Huizhou, Guangdong Province, China. J. Asia-Pacific Entomol. 22, 728–732. 10.1016/j.aspen.2019.05.009

[B74] Wang X. Xiang X. Yu H. Liu S. Yin Y. Cui P. (2018c). Monitoring and Biochemical Characterization of Beta-Cypermethrin Resistance in *Spodoptera Exigua* (Lepidoptera: Noctuidae) in Sichuan Province, China. Pestic. Biochem. Physiol. 146, 71–79. 10.1016/j.pestbp.2018.02.008 29626995

[B75] Wee C. W. Lee S. F. Robin C. Heckel D. G. (2008). Identification of Candidate Genes for Fenvalerate Resistance inHelicoverpa Armigerausing cDNA-AFLP. Insect Mol. Biol. 17, 351–360. 10.1111/j.1365-2583.2008.00809.x 18651917

[B76] Wing K. D. Sacher M. Kagaya Y. Tsurubuchi Y. Mulderig L. Connair M. (2000). Bioactivation and Mode of Action of the Oxadiazine Indoxacarb in Insects. Crop Prot. 19, 537–545. 10.1016/S0261-2194(00)00070-3

[B77] Wu S. Yang Y. Yuan G. Campbell P. M. Teese M. G. Russell R. J. (2011). Overexpressed Esterases in a Fenvalerate Resistant Strain of the Cotton Bollworm, *Helicoverpa Armigera* . Insect Biochem. Mol. Biol. 41, 14–21. 10.1016/j.ibmb.2010.09.007 20875855

[B78] Xiao Y. Liu K. Zhang D. Gong L. He F. Soberón M. (2016). Resistance to Bacillus Thuringiensis Mediated by an ABC Transporter Mutation Increases Susceptibility to Toxins from Other Bacteria in an Invasive Insect. Plos Pathog. 12, e1005450. 10.1371/journal.ppat.1005450 26872031 PMC4752494

[B79] Xu J. Su X. Bonizzoni M. Zhong D. Li Y. Zhou G. (2018). Comparative Transcriptome Analysis and RNA Interference Reveal CYP6A8 and SNPs Related to Pyrethroid Resistance in *Aedes albopictus* . Plos Negl. Trop. Dis. 12, e0006828. 10.1371/journal.pntd.0006828 30418967 PMC6258463

[B80] Yang X. Deng S. Wei X. Yang J. Zhao Q. Yin C. (2020). MAPK-directed Activation of the Whitefly Transcription Factor CREB Leads to P450-Mediated Imidacloprid Resistance. Proc. Natl. Acad. Sci. U.S.A. 117, 10246–10253. 10.1073/pnas.1913603117 32327610 PMC7229646

[B81] Zhang D.-d. Xiao X. U. Xu Y. Yang W. U. Wu Q.-l. Wu K.-m. (2021). Insecticide Resistance Monitoring for the Invasive Populations of Fall Armyworm, *Spodoptera Frugiperda* in China. J. Integr. Agric. 20, 783–791. 10.1016/S2095-3119(20)63392-5

[B82] Zhang J. Khan S. A. Heckel D. G. Bock R. (2017). Next-Generation Insect-Resistant Plants: RNAi-Mediated Crop Protection. Trends Biotechnol. 35, 871–882. 10.1016/j.tibtech.2017.04.009 28822479

[B83] Zhang L. Liu B. Zheng W. Liu C. Zhang D. Zhao S. (2020). Genetic Structure and Insecticide Resistance Characteristics of Fall Armyworm Populations Invading China. Mol. Ecol. Resour. 20, 1682–1696. 10.1111/1755-0998.13219 32619331 PMC7689805

[B84] Zhang X. Liu X. Ma J. Zhao J. (2013). Silencing of Cytochrome P450 CYP6B6 Gene of Cotton Bollworm (*Helicoverpa Armigera*) by RNAi. Bull. Entomol. Res. 103, 584–591. 10.1017/S0007485313000151 23590813

[B85] Zhao C. Tang T. Feng X. Qiu L. (2014). Cloning and Characterisation of NADPH-dependent Cytochrome P450 Reductase Gene in the Cotton bollworm,Helicoverpa Armigera. Pest Manag. Sci. 70, 130–139. 10.1002/ps.3538 23512641

[B86] Zhao J. Liu N. Ma J. Huang L. Liu X. (2016). Effect of Silencing CYP6B6 of *Helicoverpa Armigera* (Lepidoptera: Noctuidae) on its Growth, Development, and Insecticide Tolerance. J. Econ. Entomol. 109, 2506–2516. 10.1093/jee/tow181 27591286

[B87] Zhao P. Xue H. Zhu X. Wang L. Zhang K. Li D. (2022). Silencing of Cytochrome P450 Gene CYP321A1 Effects Tannin Detoxification and Metabolism in *Spodoptera Litura* . Int. J. Biol. Macromolecules 194, 895–902. 10.1016/j.ijbiomac.2021.11.144 34843814

[B88] Zhao Y.-X. Huang J.-M. Ni H. Guo D. Yang F.-X. Wang X. (2020). Susceptibility of Fall Armyworm, Spodoptera Frugiperda (J.E. Smith), to Eight Insecticides in China, with Special Reference to Lambda-Cyhalothrin. Pestic. Biochem. Physiol. 168, 104623. 10.1016/j.pestbp.2020.104623 32711763

