# Supplementary material for: Down-Regulation of P450 Genes Enhances Susceptibility to Indoxacarb and Alters Physiology and Development of Fall Armyworm, Spodoptera frugipreda (Lepidoptera: Noctuidae)
Source: Front Physiol. 2022 May 9;13:884447. doi: 10.3389/fphys.2022.884447 (PMC9125154; doi:10.3389/fphys.2022.884447)
Supplement: Supplementary file 1 [file DataSheet1.pdf]

Down-regulation of P450 genes enhances susceptibility to indoxacarb and alters physiology and development of fall armyworm, *Spodoptera frugipreda*

Table S1. List of primers used in this study for qRT-PCR

Primers used for qRT-PCR	Accession number	Sense primers	Anti-sense primers	Size of the amplicon
CYP4L4	MN480659.1	CTCCACTGTCTCTCCAAGCG	TCATGGTAAGTCGGGTCCCT	101
P450 4c3-like	XM_035586511.1	ACTGCTAGCAACCAATGCCT	AGTGTGAACGCAGGACTCAG	134
CYP4G74	KC789745.1	CCCGGACCTGCCATTATACC	ACACTCTGACTACGTTGCCG	121
CYP4G15-like protein	MN480657.1	GGAACCTACAAGGTCCACCG	AGCGCTGAAGGGTATGTAGC	123
CYP709B2-like	XM_035577862.1	GGTGACGTCATAGTGCCACA	GGCAGGAATCGTTCCGGTAT	113
CYP341A11	KC789756.1	CATCTGGCGTCGTCGTAGAA	CCGCGAACACATGGACAAAG	80
CYP4C1-like	XM_035586476.1	CCGAACCACGACGACACATA	ATCTGGGCCCCATTGTCTTG	112
CYP4d2-like	XM_035575349.1	GAGTCACCGCAAGTTCCTGA	ACATTATCAAGGGCCGCCAA	168
CYP321A9	KC789752.1	GACCCAGAAGTGTTCGACCC	TGCACTTGTAGCTTGGCGTA	125
CYP6AN4	KC789748.1	GCGCAAGTGTTCGTGTTCTT	GTACGCCAGCTGATGTAGCA	81
CYP6AE43	KJ671575.1	TGCCTTCGGAGTGGAGTCTA	TGGCCATGCAGCTCTACAAA	127
CYP337B5	KJ671580.1	TTGGGAAGCAGTCTATGCCG	GTCGTCAGGGTGCTTGAGAA	119
CYP9A59	KJ671578.1	AAGTTCGATCCGGAGCGTTT	AGGCCATCACCTTCAACTCG	133
CYP321A7	KC789750.1	AAAACAACCCCAAGACCCGT	TGAGTTCGTTCCAATGCCGA	101
CYP6AB12	KC789747.1	AGACGAGCTGTACTGGAGGT	CGTATGAGTAGCTCTGGCCG	78
CYP321B1	KC789754.1	TACGGAGGGAAGCTGACGTA	ACAGAGTCTTCCACGCACTG	125
CYP321A10	KC789753.1	CTTGGTATCACCACGTCCCC	TGTCCCAAACCGATGACTGG	84

CYP6B50	KC789749.1	AGGAGCTGCCTTGGTCAAAA	GGGAGTGAGGAGTAGTCGCA	141
CY321A8	KC789751.1	CCCAAGACGCGTGATGGTAA	ACTCTGACCCAATGCCGAAG	
CYP340L1	KC789755.1	ACATGTGCCTTGTCGCTGTA	ATCCTGCGAATGCATAGGGG	127
CYP9e2-like	XM_035584152.1	TGGACCCTGAACACTACCCA	TCTAGGGCCAACACCAAACG	113
CYP6B4-like	XM_035592007.1	TGCCTTCGGAGTGGAGTCTA	TGAAGCCCCGAAATCTCGAC	101
CYP306A1	MN480676.1	GGTGCCACGAAATGGGAGTA	GTGAGTTTGAGCTTGACCGC	116
CYP307A1	MN480677.1	ATTAGGGCAGAGTTGGACGC	GCTACGTGAGGTACGATGGG	137
CYP18A1-like	XM_035581032.1	CATCGAAGCGGTGCTCATTG	GGCTCCATGTAGATGTCGGG	171
CYP305A1	MN480675.1	TGTGGCAGGCGAACGTATAA	TTAACATGCCTCCGGCAACT	98
CYP303A1	XM_035603181.1	GGAGGATATTGCCAGGTCGG	ATCATCGTCCACAGCGTGTT	141
CYP18a1-like	XM_035581032.1	GCGGACTTGGGGTTATCACA	TGTGTTTGGCCAGAGCTTGA	104
CYP15C1	XM_035589476.1	ACTTGTTGGAGGCCGGTATG	CTAAGCGCAGGAGGTCGTAG	142
CYP301A1	XM_035582263.1	CCGGACTGCAGTTCTGGAAA	ACCAACAGATTGTTCGGCCT	119
CYP302A1	MN480674.1	TCCGTCAAGACCATCGGTTC	GCTTCTCAGCCTCCACCATT	134
CYP49A1	MN480673.1	CACCCCGACTTAGTGTTCCC	TAGACCAGGCTCATCCCCAA	150
CYP314A	MN480678.1	ATCTGCGGCGTCACTTAACA	ACGCGTTCACTAACTCCAGG	108
P450 315a1	XM_035584036.1	ACAACTGAGGCGAAGAAGGG	AGCATCACTGTGTGCGCTAA	132
P450 12B1	XM_035583496.1	AGGAGGCAAATCCATTGCGA	CTTTTCCCCTTGTGCGGTTG	113

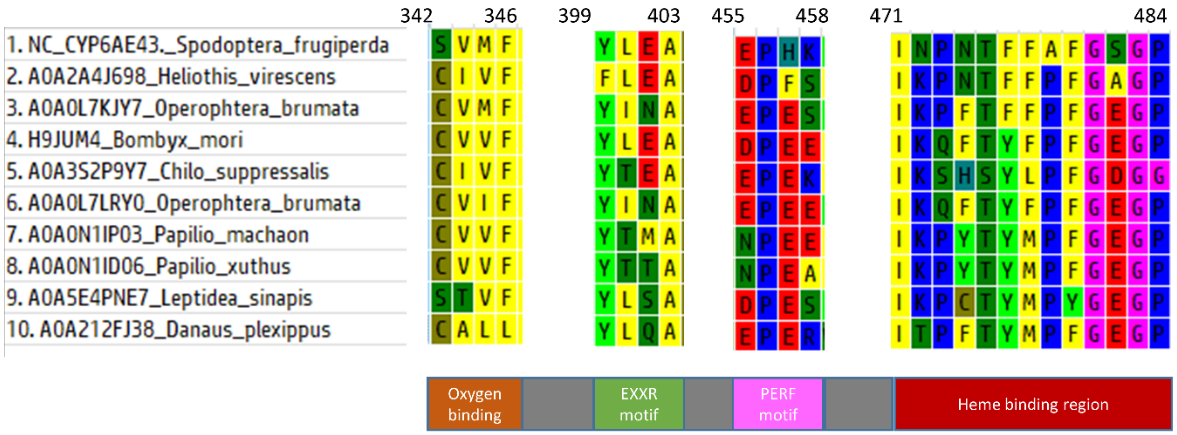

Fig. S1 P450 protein sequences and the conserved motifs of CYP5AE43 of *Spodoptera frugiperda* and other insects

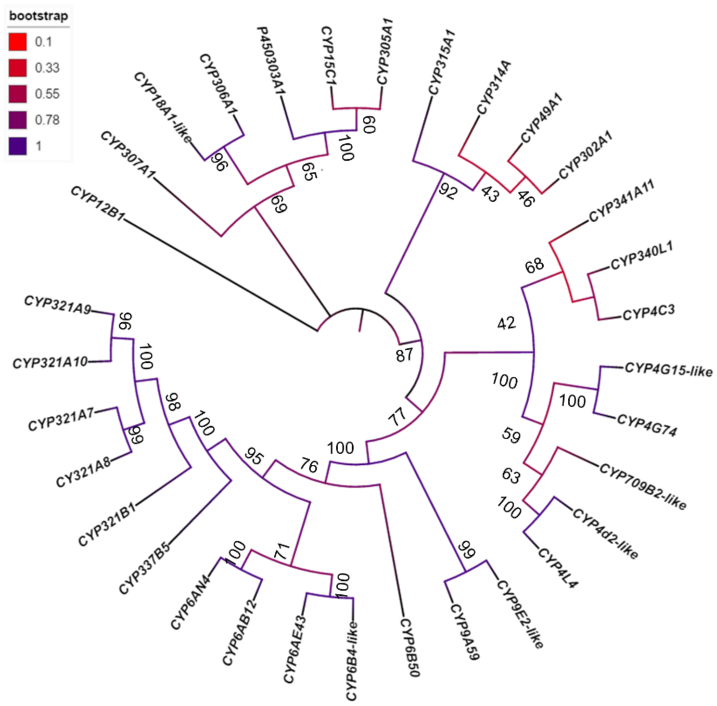

Fig. S2. Phylogenetic analysis (A) of *S. frugiperda* P450 genes belongs to CYP2, CYP3, CYP4 and Mito clad families. ClustalW was used to align protein sequences, and the phylogenetic tree was generated by neighbor-joining method with 1000 bootstrap replicates using MEGA 7 software. The genes names with accession number for constructing the tree are listed in **Table 1**.

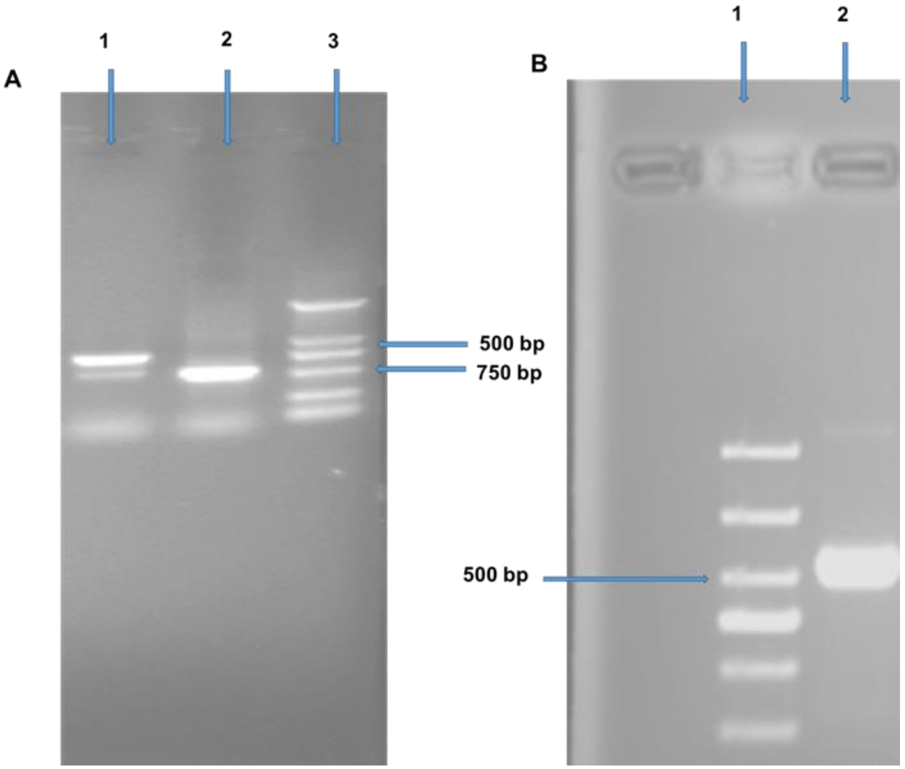

Fig. S3A shows the dsRNA of P450 gene fragment in lane 1 (dsCYP321A7) and 2 (dsCYP6AE43) and while lane 3 shows 2000 DNA marker (Sangon Biotech) while (S3B) is showing dsRED in lane 2 and 2000 bp DNA marker in lane 1.
